# Viscoelastic characterization of the human osteosarcoma cancer cell line MG-63 using a fractional-order zener model through automated algorithm design and configuration

**DOI:** 10.1038/s41598-025-16174-3

**Published:** 2025-08-26

**Authors:** Grecia C. Duque-Gimenez, Daniel F. Zambrano-Gutierrez, Maricela Rodriguez-Nieto, Jorge Luis Menchaca, Jorge M. Cruz-Duarte, Diana G. Zárate-Triviño, Juan Gabriel Avina-Cervantes, José Carlos Ortiz-Bayliss

**Affiliations:** 1 School of Engineering and Sciences, Tecnologico de Monterrey, 64700 Monterrey, Nuevo León Mexico; 2 Centro de Investigación en Ciencias Físico Matemáticas, Facultad de Ciencias Físico Matemáticas, Universidad Autónoma de Nuevo León, 66450 San Nicolás de los Garza, Nuevo León Mexico; 3 Université de Lille, CNRS, Inria, Centrale Lille, UMR 9189 CRIStAL, 59000 Lille, France; 4 Laboratorio de Inmunología y Virología, Facultad de Ciencias Biológicas, Universidad Autónoma de Nuevo León, 66450 San Nicolás de los Garza, Nuevo León Mexico; 5 Telematics Research Group, Department of Electronics Engineering, University of Guanajuato, 36885 Salamanca, Guanajuato, Mexico; 6 Secretaría de Ciencia, Humanidades, Tecnología e Innovación, 03940 Alcaldía Benito Juárez, Ciudad de Mexico, Mexico

**Keywords:** Fractional-order zener model, Viscoelasticity, Atomic force microscopy, Cells, Hyper-heuristic, Metaheuristic, Automated algorithm design, Computer science, Biomedical engineering

## Abstract

Understanding the viscoelastic properties of cells is essential for studying their mechanical behavior and identifying disease-related biomechanical markers. This paper proposes an integrated framework that combines fractional modeling with automated algorithm design to fit force-relaxation data acquired through atomic force microscopy. We employ the fractional-order zener model to describe cell relaxation curves and formulate the parameter estimation as a black-box optimization problem. To solve it, we implement a Randomized Constructive Hyper-Heuristic with Local Search (RCHH-LS) that automatically generates tailored metaheuristics (MHs) by exploring combinations of search operators. Our results show that the best-performing MH, composed of two swarm-based dynamics and a local random-walk operator ($\text {MH}_{*}^3$), achieves a performance of $3.00\times 10^{-3}$, representing a 75% improvement over the mean of all candidate configurations. Subsequent hyperparameter tuning with Optuna reduces this value to $2.86\times 10^{-3}\pm 2.43\times 10^{-7}$, a further 4.7% gain relative to the untuned version while preserving high stability and repeatability. In an evaluation of 21 instances (force-relaxation curves), the tuned $\text {MH}_{*}^3$ provided the best result in 19 cases, achieving an average of $3.31\times 10^{-3}$, about 12% better than the best two-operator alternative and a coefficient of variation below 0.01%, underscoring its generalization capability. The FOZ model fitted using this solver generalizes well to independent datasets and captures critical viscoelastic parameters. We also confirm that $E_1$, $\tau$, and $\alpha$ are sensitive to the applied force via a statistical analysis, while $E_0$ remains stable, reinforcing its association with intrinsic cell properties. These results highlight the effectiveness of combining fractional viscoelastic modeling with automated MH design for robust and interpretable mechanical characterization of cells. The proposed approach reduces manual intervention, ensures generalizability, and offers a scalable solution for computational biomechanics.

## Introduction

The study of diseases such as cancer, which remains one of the leading causes of mortality worldwide, requires the development of advanced techniques that contribute to its diagnosis and comprehensive characterization^1,2^. Beyond the genetic and biochemical aspects traditionally addressed, the mechanical properties of cells have proven to be pivotal elements in understanding their behavior in different physiological and pathological contexts^3–5^. These properties are critical in the migration, differentiation, and proliferation processes^6–8^. Research has shown that changes in mechanical behavior can serve as disease markers^9–13^, including cancer, where increased deformability facilitates dissemination^14–17^. In this sense, developing accurate methodologies to measure and analyze these properties is crucial to advancing biomedicine and cellular biomechanics. Cells are viscoelastic materials^18^, since they exhibit a behavior between elastic solids and Newtonian fluids. There are several tests for quantifying the viscoelastic response of these materials. Among these are the relaxation test, in which a constant deformation is applied and the stress response is monitored; the creep test, where a constant stress is applied and the resulting deformation is measured; and oscillatory tests, which apply an oscillatory load and track the induced deformation^19^.

In addition to these mechanical tests, a variety of experimental techniques have been developed to characterize the viscoelasticity of biological samples, including atomic force microscopy (AFM), Optical Tweezers (OT), Magnetic Tweezers (MT), Micropipette Aspiration (MPA), and microfluidic methods^20^. Each technique offers distinct advantages in terms of spatial resolution, measurement sensitivity, and the type of mechanical load applied. Among these, AFM stands out due to its nanometer-scale spatial resolution, picoNewton force sensitivity, and capability to analyze live cells under near physiological conditions. AFM gained particular relevance in the study of cell mechanics when it was shown that cancer cells could be distinguished from normal cells based on their Young’s modulus, which was found to be an order of magnitude lower in cancer cells^21^. Since then, it has become one of the most widely used techniques for the characterization of biological samples^22^ and has demonstrated great potential in biomedical applications^23^. Furthermore, AFM enables the mapping of local mechanical properties and supports various mechanical testing modalities, such as relaxation, creep, and oscillatory tests, depending on the system’s hardware configuration^22,24^. To quantitatively describe the viscoelastic responses observed in creep or relaxation experiments, mathematical models that employ mechanical analogies formed by springs (Hooke’s law) and dampers (Newton’s law) are commonly used. Among them, models such as the Maxwell model^25^, the Kelvin-Voigt model^26^, and the Standard Linear Solid (SLS or Zener)^27^ stand out. However, these models present limitations in reproducing the power-law behavior and long-term memory effects that often characterize biological materials^28,29^; moreover, they only allow describing monoexponential relaxation functions^30^. One way to circumvent this drawback is to concatenate multiple Maxwell or Kelvin-Voigt branches, but this complicates the physical interpretation by considerably increasing the number of fitting parameters^31^. To address these limitations, fractional models, based on derivatives of non-integer order, have emerged as powerful alternatives^3,28,32,33^. Fractional models naturally account for both power-law dynamics and memory effects, making them particularly well-suited for modeling the complex mechanical behavior of biological systems^32,33^. Whether classical or fractional models are employed, these models require parameter estimation through a fitting process. This task involves fitting the model response to match the experimental data, usually by minimizing an error metric that quantifies the difference between observed and predicted behavior. In most cases, this leads to an optimization problem defined over a continuous and often unknown solution space. Therefore, identifying the parameters and their adequate values becomes challenging, especially regarding experimental variability and the intrinsic model complexity; these are the well-known ill-posed inverse problems^34^. In this sense, heuristic-based algorithms, like metaheuristics (MHs), are versatile because they can explore complex search spaces without requiring gradient information or convexity requirements^35^. MHs such as Particle Swarm Optimization, Genetic Algorithms, Differential Evolution, and Simulated Annealing have demonstrated efficacy in various practical engineering scenarios^36^. The selection of a suitable Metaheuristic (MH) for a given optimization problem remains a non-trivial challenge due to the increasing number of available algorithms and the complexity involved in tuning their hyperparameters^37^. Furthermore, the No-Free-Lunch theorem stresses that no algorithm can optimally solve all possible problems, implying that the suitability of the algorithm is intrinsically dependent on the problem^38^. In other words, when considering the entire space of possible problems, all algorithms perform equally on average. Therefore, an algorithm can only outperform others when applied to particular subsets of problems. In addition, many MHs in the literature are constructed primarily from metaphors rather than based on mathematical foundations^39,40^. Because of this, the scientific community’s attention has recently shifted to Automated Algorithm Design and Configuration (AADC) frameworks^41–44^. These approaches allow researchers to automatically select or adjust MHs by building or adapting a custom solver^37^. One strategy to address AADC is to employ Hyper-Heuristics (HHs)^44^. These operate at a higher level of abstraction by selecting, combining, or generating heuristics. This paradigm enables the automatic configuration of MHs tailored to specific problem domains using Simple Heuristics (SHs) or Search Operators (SOs)^45^.

In this work, we propose an integrated framework that combines Fractional-Order (FO) viscoelastic modeling and the automated design and configuration of MHs through an HH approach to characterize the mechanical behavior of MG-63 osteosarcoma cells. Specifically, we employ the Fractional-Order Zener (FOZ) model to describe the force-relaxation curves obtained by AFM and formulate the parameter estimation process. To solve this, we implement a Randomized Constructive Hyper-Heuristic with Local Search (RCHH-LS) that automatically generates tailored MHs from a predefined set of SOs. Additionally, a hyperparameter tuning process using the Optuna framework^46^ further refines the best-performing MH, maximizing fitting performance and ensuring high model accuracy, while also increasing its generalizability.

The main contributions of this work are the following:

- We present a physically consistent formulation of the FOZ model using the Caputo derivative. We validate its ability to accurately describe the relaxation curves obtained from MG-63 cells under different mechanical loading conditions.
- We implemented an RCHH-LS to automatically generate tailored MHs (customized MHs for the FOZ model), thereby solving the parameter estimation problem.
- We gather statistical evidence that different configurations of MHs, even those initially associated with low performance, can be improved through an adequate hyperparameter tuning process.
- We provide, to our knowledge, the first study of how the applied loading force influences the FOZ-derived viscoelastic parameters. Our analysis reveals that $E_{0}$ is the parameter that exhibited the least variation across force regimes, while $E_{1}$, $\tau$, and $\alpha$ vary significantly with force magnitude.

The remainder of this document is as follows. Section “Foundations” introduces the core concepts related to our work, such as fractional calculus, viscoelastic models, metaheuristics, and automated algorithm design. Section “Proposed approach” describes integrating AFM experiments with algorithm configuration. Section “Methodology” outlines the experimental setup and the hyper-heuristic framework. Section “Experimental results” presents and analyzes the results obtained. Finally, Section “Conclusion” summarizes the main findings and discusses future research directions.

## Foundations

This section presents the theoretical background that supports our methodology, including an overview of fractional calculus, viscoelasticity, cell mechanical characterization techniques, the FOZ model, metaheuristics, and automated algorithm design and configuration.

### Fractional calculus

Fractional Calculus (FC) provides a structure for describing derivatives and integrals of non-integer order, all represented by an integro-differential operator of order $\alpha$^47^, denoted as $_{t_0} \mathscr {D}_t^{{\alpha }}$. This parameter $\alpha$ defines the FO, allowing one to work with simultaneous derivation and integration in the same function $f$, which is formulated in terms of $\Re (\alpha )$ as follows:

1 $$\begin{aligned} _{t_0} \mathscr {D}_t^\alpha f(t)=\text {H}(\Re (\alpha )) \frac{d^\alpha }{d t^\alpha } f(t)+\text {H}(-\Re (\alpha )) \int _{t_0}^t f(\tau )(d \tau )^{-\alpha }+(1-\text {H}(|\Re (\alpha )|)) f(t), \end{aligned}$$

where $\Re \{\cdot \}$ is the real part function, and $t_{0}$ is the initial operating point and *t* is the independent variable assumed dimensionless for this particular case. Lastly, $\text {H}(\cdot )$ is the Heaviside function that selects the appropriate operation based on $\Re (\alpha )$. For the special case $\alpha = 0$, we adopt the convention $\text {H}(0) = 0$, in which case the fractional operator reduces to the identity $_{t_0} \mathscr {D}_t^{0}f(t)=f(t)$.

The literature offers several approaches to FO differentiation and integration, each offering advantages for specific engineering or science applications. Among the most widely used in the literature and practical implementations are the Grünwald-Letnikov (G-L)^48^, Riemann-Liouville (R-L)^49^, and Caputo^50^ fractional operators, which have successfully described many applications. This paper adopts the Caputo derivative, which provides a natural framework for incorporating integer-order initial conditions. Besides, it is particularly suitable for modeling physical systems with well-defined initial states. Caputo’s formulation corresponds to a variation of the R-L expression designed to facilitate the integration of initial conditions in boundary value problems^50^, and it is formally defined as

2 $$\begin{aligned} {}^{\text {C}}_{{t}_0}{\mathscr {D}_t^{{\alpha }}} f(t)=\frac{1}{\Gamma (n-{{\alpha }})} \int _{t_0}^t (t-\tau )^{n-{{\alpha }}-1}\left. \frac{d^n}{dt^n}f(t)\right| _{{t}=\tau } d \tau , \end{aligned}$$

where ${\alpha }$ is the fractional order, $\Gamma (\cdot )$ is the Gamma function and $n=\lfloor {{\alpha }}\rfloor$ since ${{\alpha }}\in \mathbb {R}_+$^47^.

Recall the Laplace transform of *f*(*t*) is defined as follows,

3 $$\begin{aligned} F(s)\triangleq \mathscr {L}\{f(t)\}(s)=\lim _{\tau \rightarrow \infty }\int _{t_0}^{\tau } e^{-st} f(t)dt, \end{aligned}$$

where $s = \rho + \text {i} \omega$ represents the complex variable comprising the real parts $\rho$ and $\omega$. Additionally, the Laplace transform of the *n*th-order derivative generalized under continuity conditions on $\mathbb {R}_+$ is expressed as shown,

4 $$\begin{aligned} \mathscr {L}\left\{ \frac{d^n}{dt^n}f(t)\right\} (s)=s^n F(s) - \sum _{k=1}^{n} s^{n-k}\left. \frac{d^{k-1}}{dt^{k-1}}f(t)\right| _{t=t_0}. \end{aligned}$$

According to^47^, the Laplace transforms of FO derivatives can be similarly defined via,

5 $$\begin{aligned} \mathscr {L}\left\{ {}^{\text {C}}_{{t}_0}{D_t^{{\alpha }}} f(t)\right\} (s)= s^{{{\alpha }}} F(s)-\sum _{k=0}^{n-1}s^{{{\alpha }}-k-1} \left. \frac{d^k}{dt^k}f(t)\right| _{t=t_0}. \end{aligned}$$

This transformation gives us insight into the behavior of FO systems, opening research opportunities for new analytical and numerical methods to address complex problems in science and engineering.

### Viscoelasticity

Viscoelastic materials are those that exhibit both elastic and viscous behavior. These materials possess a time-dependent response when subjected to constant strain or stress. So, when subjected to constant strain ($\epsilon (t) = \epsilon _{0}$), they experience *relaxation*, whereas under constant stress ($\sigma (t) = \sigma _{0}$ [Pa]), they exhibit *creep*. These responses can be described using the relaxation function or the creep function, respectively, which are defined as^19^:

6 $$\begin{aligned} E_{\text {rel}}(t) = \frac{\sigma (t)}{\epsilon _0},\quad \text {and}\quad J_{\text {creep}}(t) = \frac{\epsilon (t)}{\sigma _0}. \end{aligned}$$

Mechanical arrangements consisting of springs, representing the elastic part, and dashpots, representing the viscous part, are commonly used to model the time-dependent response of viscoelastic materials. The simplest models for describing viscoelastic behavior are the Maxwell model, which consists of a spring and a dashpot connected in series, and the Kelvin model, which includes a spring connected in parallel to a dashpot. The Maxwell model describes fluid-like materials, while the Kelvin model represents solid-like materials^51^. However, the complexity of cells demands arrangements with more elements, including fractional-order elements, to obtain a good representation of their viscoelastic response^52^.

### Cell mechanical characterization techniques

Both cancerous and normal cells exhibit viscoelastic properties that give rise to time-dependent responses such as stress relaxation, creep, strain-rate sensitivity, and hysteresis^53^. Quantifying these behaviors requires devices capable of applying and measuring forces ranging from the piconewton to the micronewton scale with micrometer and nanometer resolution. Among the widely used techniques are Atomic Force Microscopy (AFM), Optical Tweezers (OT), Magnetic Tweezers (MT), Particle-Tracking Microrheology (PTM), and Micropipette Aspiration (MPA)^20^. AFM indents the cell surface with a nanometer-scale probe located at the end of a cantilever; a laser beam is directed onto the back side of the cantilever and reflected toward a photodetector. The cantilever deflection, measured by the photodetector, is then converted into force, enabling the generation of force curves^54,55^. OT traps a dielectric bead at the focus of a tightly focused laser beam, where the optical gradient force acts as a calibrated linear spring in the piconewton range^55,56^. MT tether paramagnetic microbeads to the sample and apply tensile or torsional loads via a controlled magnetic field; bead displacement, recorded optically in real time, reveals the deformation of the attached structure^55^. PTM tracks the Brownian motion of tracer particles embedded in the cytoplasm and converts their mean-square displacement into the complex shear modulus of the intracellular medium^57^. Finally, MPA partially draws a cell into a glass microcapillary under known negative pressure, and the aspirated length-pressure relationship provides estimates of cortical tension or an effective Young’s modulus^58^. Each method offers advantages by combining different ranges of sensitivity, temporal resolution, and throughput. The principal advantages and limitations of these techniques are summarized in Table 1.

**Table 1:** Advantages and limitations of single-cell mechanical characterization techniques.

Technique	Main advantages	Main limitations
AFM	Sub-nanometre spatial resolution; broad force range (pN-$\mu$N); local mechanical mapping of living cells.	Tip-sample contact may perturb the specimen; requires precise cantilever calibration^54,55^.
OT	Non-contact manipulation; piconewton force sensitivity; high temporal resolution (kHz).	Limited to dielectric beads or cells in suspension; maximum force $\lesssim 200$ pN; potential photothermal heating^55,56^.
MT	Simultaneous force and torque application; long-term stable loading; negligible heat generation.	Requires bead functionalisation; spatial resolution limited by optical tracking; mainly suited to tethered samples^55^.
PTM	Passive and minimally invasive; probes intracellular rheology over a wide frequency range; parallel measurements possible.	Delivers relative rather than absolute moduli; tracer size and local heterogeneity complicate interpretation^57^.
MPA	Simple analytical framework; yields whole-cell mechanical parameters; applicable to adherent or suspension cells.	Coarse spatial resolution; quasi-static measurement; possible membrane damage at high suction^58^.

Among the techniques summarized in Table 1, AFM combines exceptional versatility, nanometre-scale spatial resolution, and the widest usable force range, making it the extensively adopted device for single-cell nanomechanics^13^. Moreover, the versatility of AFM enables various mechanical tests to characterize the viscoelastic properties of cells, including loading-unloading, stress relaxation, and creep^22,24^. While classical mechanical models have traditionally been used to analyze these tests, fractional models, such as the fractional-order Zener model, have shown superior performance, particularly in relaxation and creep experiments, by more accurately capturing the viscoelastic behavior of cells^52,59,60^.

### Fractional-order zener model

**Fig. 1:**
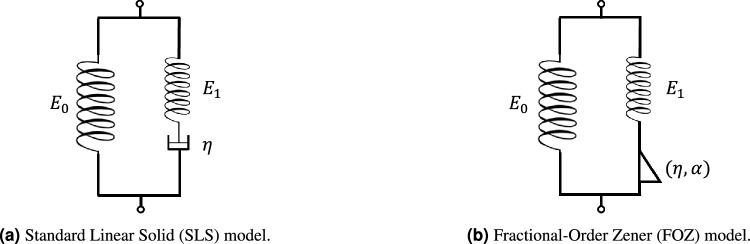
The schematic representation of mechanical models: (a) Standard Linear Solid (SLS), and (b) Fractional-Order Zener (FOZ), which are composed of springs, dampers, and spring-dampers.

The Standard Linear Solid (SLS) model, also known as the Zener model, is a traditional viscoelastic framework formed by a spring with a Maxwell component in a particular arrangement, which here is a spring and a damper in a series configuration, as Fig. 1 depicts. The differential equation describing this behavior is expressed as,

7 $$\begin{aligned} {\dot{\sigma }(t)}+{\frac{E_1}{\eta }}{\sigma (t)} = {(E_1 + E_0)} {\dot{\varepsilon }(t)} + {\frac{E_1 E_0}{\eta }}{\varepsilon (t)}, \end{aligned}$$

where $E_{0}$ [Pa] and $E_{1}$ [Pa] represent the springs elastic moduli, and $\eta$ [Pa$\cdot$s] corresponds to the dashpot viscosity.

This work considers the stress relaxation, simplifying the constitutive differential equation to

8 $$\begin{aligned} \dot{\sigma }(t) + \frac{E_1}{\eta } \sigma (t) = \frac{E_1 E_0}{\eta } \varepsilon _0. \end{aligned}$$

Recall that, in a relaxation test, the strain $\varepsilon (t)$ is maintained constant, i.e., $\varepsilon (t)=\varepsilon _{0}$. Now, to transition to the fractional calculus and introduce the FOZ model (see Fig. 1), we must consider that including fractional derivatives on the model as is produces unit inconsistency^61^. Therefore, the differential model (8) must be nondimensionalized by identifying the system’s critical parameters, defined as

9 $$\begin{aligned} \hat{\tau } = t / t_{c}\quad \text {and}\quad \varphi = \sigma / \sigma _{c}, \end{aligned}$$

since $t_{c} = \eta / E_{1}$ and $\sigma _{c} = \varepsilon _{0} E_{0}$. The resulting dimensionless model is given by

10 $$\begin{aligned} \frac{d\varphi }{d\hat{\tau }} + \varphi = 1, \end{aligned}$$

where we can incorporate the fractional Caputo operator. This gives place to the proposed fractional-order differential equation as follows,

11 $$\begin{aligned} {}^{C}_{0}{D}_{\hat{\tau }}^\alpha \varphi (\hat{\tau }) + \varphi (\hat{\tau }) = 1, \quad 0 < \alpha \le 1. \end{aligned}$$

The unconventional differential equation in (11) is solved by applying the Laplace transform for the Caputo derivative (5), providing the expression in the *s*-domain such as

12 $$\begin{aligned} \mathscr {L} \left\{ {}^{C}_{0}{D}_t^\alpha \varphi (\tau ) \right\} = s^\alpha \tilde{\varphi }(s) - s^{\alpha -1} \varphi (0), \end{aligned}$$

which can be rewritten as,

13 $$\begin{aligned} \tilde{\varphi }(s) = \frac{1}{s(s^\alpha + 1)} + \varphi (0) \frac{s^{\alpha -1}}{s^\alpha + 1}, \end{aligned}$$

since $\varphi (0) = 1 + {E_1}/{E_0}$ is obtained from $\varphi (0) = \sigma (0) / \sigma _c$ and the initial stress $\sigma (0) = (E_0 + E_1)\,\varepsilon _0$.

The temporal solution can be obtained with ease by applying the inverse Laplace transform $\mathscr {L}^{-1}\left\{ \tilde{\varphi }(s) \right\}$ and then reintroducing the critical parameters to dimensionalize the system, as shown

14 $$\begin{aligned} \varphi (\tau ) = 1 + (\varphi (0) - 1) \xi _{\alpha }(-\tau ^\alpha ) \Longrightarrow \frac{\sigma (\tau )}{\sigma _c} = 1 + \frac{E_1}{E_0} \xi _{\alpha } \left\{ -\left( \frac{\tau }{t_c} \right) ^\alpha \right\} \Longrightarrow {\sigma (t)} = E_0\varepsilon _0 + {E_1}\varepsilon _0 \xi _{\alpha } \left\{ -\left( \frac{E_1 t}{\eta } \right) ^\alpha \right\} , \end{aligned}$$

where $\xi _{\alpha }(\cdot )$ is the one-parameter Mittag-Leffler function. Finally, the relaxation function is then obtained through the expression in (6), as follows,

15 $$\begin{aligned} E_{\text {rel}}(t) = E_0 + {E_1} \xi _{\alpha } \left\{ -\left( \frac{E_1 t}{\eta } \right) ^\alpha \right\} . \end{aligned}$$

Regarding this relaxation model achieved above, it is paramount to determine the viscoelastic parameters $E_{0}$, $E_{1}$, $\alpha$, and $\eta$ to accurately characterize the mechanical behavior of the cells where the ratio $E_{1}/\eta$ is generally related to the relaxation time $\tau$. Estimating these parameters involves solving an optimization problem to minimize the discrepancy between the theoretical relaxation function and experimental data.

### Optimization problem

An optimization problem consists of finding an optimal solution $\vec {x}_{*}$ within a search space $\mathfrak {X}$, such that an objective function $f:\mathfrak {X} \rightarrow \mathbb {R}$ provides the best possible output. Depending on the problem, the optimization task can be formulated as either a minimization or maximization problem^62^:

16 $$\begin{aligned} \vec {x}_{*}=\underset{\vec {x} \in \mathfrak {X}}{\arg \min }\left\{ f(\vec {x})\right\} \quad \text {or} \quad \vec {x}_{*}=\underset{\vec {x} \in \mathfrak {X}}{\arg \max }\left\{ f(\vec {x})\right\} , \end{aligned}$$

In the minimization case, the optimal solution $\vec {x}_{*}^{} \in \mathfrak {X}$ satisfies $f(\vec {x}_{*}^{}) \le f(\vec {x})$ for all $\vec {x} \in \mathfrak {X}$. Similarly, in maximization, we have $f(\vec {x}_{*}) \ge f(\vec {x})$ for all $\vec {x} \in \mathfrak {X}$. For simplicity, we refer to a minimization problem using the tuple $(\mathfrak {X}, f)$, which simultaneously represents its domain and objective function.

### Metaheuristics

A Metaheuristic (MH) can be defined as an iterative optimization procedure designed to approximate an optimal solution $\vec {x}_{*}$ for a given optimization problem, driven by an objective function $f(\vec {x})$. Some classic examples of MHs include Simulated Annealing (SA) and Genetic Algorithms (GAs), each inspired by distinct natural processes. On the one hand, SA^63^ simulates the physical annealing process in metallurgy, where a material is heated and then slowly cooled to achieve a low-energy crystalline structure. This MH uses a random search to perturb the current solution and applies the Metropolis criterion to probabilistically accept worsening moves, thereby avoiding premature convergence. On the other hand, GA^64^ draws inspiration from the principles of natural selection and genetics. It maintains a population of candidate solutions that evolve over generations through crossover and mutation operators, guided by selection mechanisms that favor fitter individuals.

Both methods illustrate how biologically or physically motivated strategies can be abstracted into a structured search process over complex optimization landscapes. Nevertheless, these and many other MHs are structured sequences of Simple Heuristics (SHs)^65^ executed iteratively until fulfilling a stopping criterion. Formally, an MH can be represented as^44^:

17 $$\begin{aligned} \text {MH}_o \triangleq \left\langle h_{i}, h_{o}, h_{f}\right\rangle =h_f\left( h_o\right) \circ h_{i}, \end{aligned}$$

where $h_{i}$ denotes an initialization heuristic, $h_{o}$ represents a Search Operator (SO) exploring the solution space. $h_{o}$ can individually comprise one or multiple SOs. For instance, several ($\varpi >1$) SOs applied in sequence yield $\displaystyle h_{o}=h_\varpi \circ h_{\varpi -1}\circ \cdots \circ h_{1}$, $\forall \,h_{k}\in \vec {h}\in \mathfrak {H}^\varpi$. In such a case, $\varpi$ represents the maximum MH cardinality and $\mathfrak {H}^\varpi$ denotes the space of available heuristics. Finally, $h_{f}$ is a finalization procedure determining the solution, and $\circ$ is the composition operator.

This modular definition allows the representation of well-established MHs in the literature and expands the possibilities of designing new search strategies in a more structured way^44^. Unlike traditional metaphor-based proposals, this approach is based on the essential MH components: initialization ($h_{i}$), search operators ($h_{o}$), and stopping criteria or finalizer ($h_{f}$)^45^. Furthermore, notice that an SO is composed of a perturbative ($h_{p}$) and a selective heuristic ($h_{s}$), i.e., $h_{o}\equiv \left( h_{s}\circ h_{p}\right)$, where $h_{p}$ modifies a candidate and $h_{s}$ evaluates a candidate solution and decides either to accept or search for another solution.

Following this approach, well-known MHs can be formally described in terms of their constituent SOs. Returning to SA and GA, these methods serve as illustrative examples that can be decomposed into sequences of perturbative and selective heuristics. SA can be expressed as $\displaystyle \text {MH}_{\text {SA}} = h_{f} \circ (h_{\text {M}} \circ h_{\text {RS}}) \circ h_{i}$, where $h_{\text {RS}}$ is a random search, $h_{\text {M}}$ is a Metropolis-based selector, and $h_f$ represents the finalization process. Similarly, GA follows the structure $\text {MH}_{\text {GA}} = h_{f} \circ (h_{\text {G}}\circ h_{\text {GM}}) \circ (h_{\text {D}} \circ h_{\text {GC}})\circ h_{i}$, where $h_{\text {GC}}$ is a genetic crossover, $h_{\text {D}}$ represents a direct selector, $h_{\text {GM}}$ is a genetic mutation and $h_{\text {G}}$ is a greedy selector.

### Automated algorithm design and configuration

Automated Algorithm Design and Configuration (AADC) has become increasingly relevant in recent years^41,42^, motivated by the need to reduce the proliferation of new MHs without solid mathematical foundations^66^. Traditionally, the development and tuning of optimization algorithms have relied on trial-and-error approaches, where experts manually adjust the algorithm parameters and components^37^. However, this strategy often leads to suboptimal configurations since the deep exploration of the space configurations is limited and highly dependent on the designer’s expertise.

An AADC problem can be rigorously formulated within the General Combinatorial Optimization Problem (GCOP)^67,68^, specifically tailored to synthesize heuristic-based algorithms. Therefore, the objective is to identify the optimal MH configuration in a predefined heuristic space that maximizes performance over a given optimization problem, such as

18 $$\begin{aligned} \text {MH}_* = \underset{\text {MH} \in \mathfrak {H}^\varpi }{\arg \max } \left\{ Q(\text {MH} \mid \mathfrak {X}) \right\} , \end{aligned}$$

where $\mathfrak {H}$ represents the heuristic space and $\varpi$ denotes the MH cardinality. The metric $Q(\text {MH} \mid \mathfrak {X})$ assesses the performance of a given candidate MH to deal with a given optimization problem $(\mathfrak {X}, f)$.

In this work, the GCOP is addressed using Hyper-Heuristics (HHs), which operate as high-level methodologies capable of automatically exploring the heuristic space to construct effective solution strategies^69^. Notice that MH and HH present fundamental distinctions in their functionality. While an MH follows a fixed algorithmic design, an HH continuously reorganizes its internal components or heuristics, refining the SOs arrangement to improve performance in diverse problem instances^70^. Furthermore, this approach is model-free^71^, focusing on building an MH by combining available SOs. Still, once the MH description is obtained, the configuration of its internal hyperparameters becomes a fundamental issue, since the MH performance significantly depends on properly tuning these parameters. Consequently, a model-based approach is used to address this problem^71^, where the objective is to tune the hyperparameters associated with this customized MH. This optimization process can be formally defined by

19 $$\begin{aligned} \Theta _* = \underset{\Theta \in {\Psi }}{\arg \max } \left\{ Q(\text {MH}, \Theta \mid \mathfrak {X}) \right\} , \end{aligned}$$

where $\Theta$ represents the set of hyperparameters, and $\Psi$ denotes the hyperparameter search space. A more comprehensive strategy involves addressing both components simultaneously: selecting the SOs and dynamically adjusting their associated hyperparameters. This unified formulation is expressed as

20 $$\begin{aligned} (\text {MH}_*, \Theta _*) = \underset{\text {MH} \in \mathfrak {H}^\varpi , \Theta \in {\Psi }}{\arg \max } \left\{ Q(\text {MH}, \Theta \mid \mathfrak {X}) \right\} , \end{aligned}$$

where Genetic Programming (GP) is a suitable solution to this problem.

## Proposed approach

We propose a three-step optimization-based approach for MH identification and configuration focused on analyzing the biomechanical properties of MG-63 cells. First, MG-63 cells are cultured under controlled conditions to ensure proper adhesion before measurement. Next, relaxation experiments are conducted using AFM, and the acquired dataset is processed to extract the force-time curves for analysis. Finally, we implement the MH automated design using Randomized Constructive Hyper-heuristic with Local Search (RCHH-LS). The achieved MH is subsequently fine-tuned to maximize its performance.

### Atomic force microscopy measurements

**Fig. 2:**
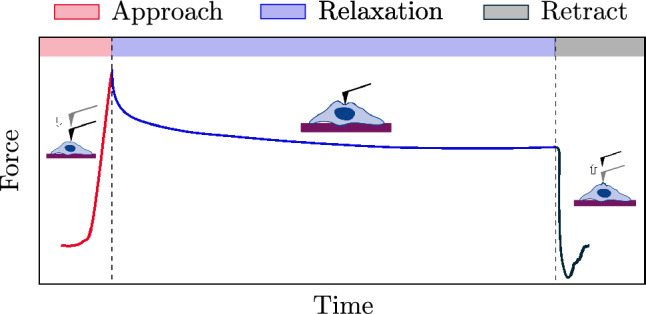
Representation of a force-time experiment using AFM. The curve shows three phases: approach (red area), relaxation (blue area), and retraction (black area).

The measurements are conducted with an NT-MDT Spectrum Instruments AFM (Ntegra model) operating in force mode. In a force-relaxation experiment, the AFM tip approaches the cell via piezoelectric extension until it reaches either a predefined maximum load or a predefined piezoelectric extension, inducing an indentation. The tip then remains in position for a set duration to capture the relaxation behavior before retracting to its original position. Figure 2 illustrates the process for generating a force-time profile composed of three phases. In the approach phase, the force increases as the tip approaches the cell. In the relaxation phase, the force decays with time, and one observes the relaxation behavior of the cell. Finally, the tip is withdrawn in the retraction phase.

In a typical AFM force-relaxation experiment, two force-related curves are recorded: force versus piezoelectric extension and force versus time. Although these curves are presented in terms of force, the raw data correspond to electrical current intensity, which is subsequently converted to force *F* [N] using Hooke’s law:

21 $$\begin{aligned} F = k \cdot d, \end{aligned}$$

since *k* [N/m] is the cantilever’s spring constant, and *d* [m] is its deflection. The deflection is assessed from the measured current intensity *I* [A] through the relation $d = sI$, where *s* [m/A] is the cantilever sensitivity factor. This sensitivity is calibrated by acquiring a force-distance curve on a rigid surface (e.g., the Petri dish) and calculating the slope of the linear contact region.

Moreover, the indentation depth $\delta$ [m] generated in the cell is estimated by subtracting the cantilever deflection *d* [m] from the piezoelectric extension *z* [m]^72^, as follows:

22 $$\begin{aligned} \delta = (z - z_0) - (d - d_0), \end{aligned}$$

where $d_0$ is the cantilever deflection at the contact point between the indenter tip and the sample, and $z_{0}$ is the corresponding piezoelectric extension at $d_{0}$.

### Randomized constructive hyper-heuristic with local search

The high-level problem, defined by GCOP in (18), is solved using the Randomized Constructive Hyper-heuristic with Local Search (RCHH-LS), which explores different SO configurations on the low-level problem. Our proposal includes a heuristic space $\mathfrak {H}$, composed of all available SOs, and building heuristic sequences to configure candidate MHs (17). Subsequently, the performance of a candidate MH is estimated using the metric defined by^45^

23 $$\begin{aligned} {Q}\bigl (\text {MH}_o \,\big \vert \, \mathfrak {X}\bigr ) \;=\; \Bigl (\operatorname {med}(F_h) \;+\; \operatorname {iqr}(F_h)\Bigr ), \end{aligned}$$

where $F_h \;=\; \bigl \{ f(\vec {x}_{r,*}) \;\big \vert \; \forall \,\vec {x}_{r,*}\in X_* \bigr \}$ is the objective function evaluated at the best solution $\vec {x}_{r,*}$ found by $\text {MH}_{o}$ in each *r*-th run, and $X_*=\{\vec {x}_{1,*},\,\ldots ,\,\vec {x}_{N_r,*}\}$ contains the solutions from $N_{r}$ runs. The operators $\operatorname {med}$ and $\operatorname {iqr}$ denote the median and the interquartile range of the values in $F_{h}$.

Pseudocode 1 presents the RCHH-LS strategy for obtaining MHs through a three-stage process executed *T* times, or HH iterations (lines 2 to 12). The first stage (lines 3 to 5) begins with the random building of a candidate MH, where up to $\varpi _{\max }$ operators are selected from the heuristic space $\mathfrak {H}$ to build a configuration $\operatorname {MH}^{(0)}$. This candidate MH’s performance is measured via $N_r$ independent runs using the EvalPerformance routine. Such a value is stored as $Q^{(0)}$. The second stage (lines 6 to 8) is similar to the previous one. Still, with a subtle variation, i.e., a local search is applied to explore the neighborhood of $\operatorname {MH}^{(0)}$. This procedure randomly chooses two actions to render a neighbor sequence: Swap and Replace. In the Swap action, two operators from the sequence are randomly selected and their positions interchanged. In the Replace move, only one operator is chosen randomly and replaced by another, drawn uniformly from the available heuristic space and not already present in the sequence. In the third stage (lines 9 to 12), after generating the neighbor configurations, the one yielding the best performance is selected and re-evaluated to obtain $Q^{(\textrm{LS})}$. Then, the best configuration between $\operatorname {MH}^{(0)}$ and $\operatorname {MH}^{(\textrm{LS})}$ is retained. The global record is updated *if* its performance is better than the current global best. Finally, the HH returns the best MH found and its performance value. This process relies on two supporting routines: EvalPerformance, which performs the statistical evaluation of each MH, and EvalMH (lines 20 to 31), which executes the MH on a population of size *N*.

**Pseudocode 1:**
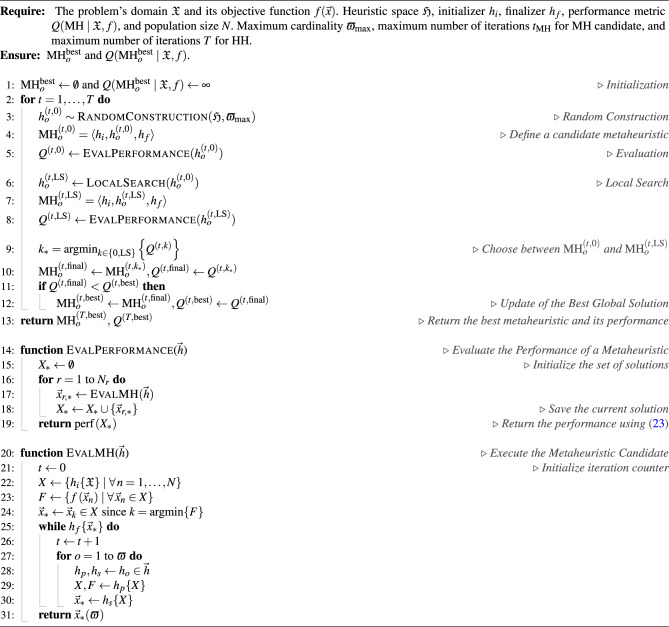
Randomized Constructive Hyper-heuristic with Local Search (RCHH-LS)

## Methodology

**Fig. 3:**
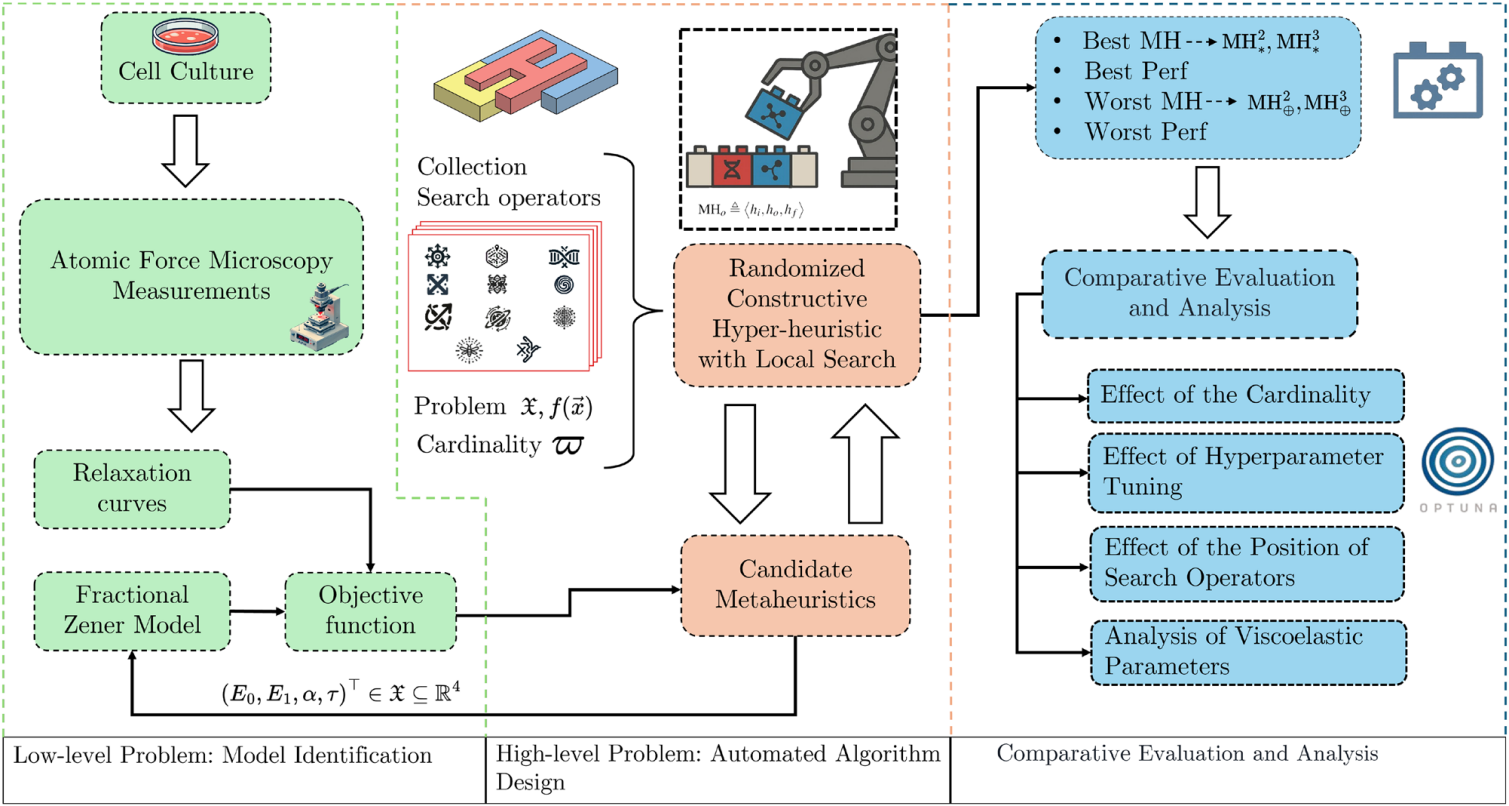
Overview of the implemented three-fold methodology, comprising, from left to right, the low-level problem (model identification), the high-level problem (automated algorithm design), and the comparative evaluation and analysis stages.

Figure 3 shows the methodology we implemented for our proposal, consisting of three main stages. The first, on the left-hand side (green area), illustrates the low-level problem, starting with cell culture and AFM measurements and ending with the obtaining of relaxation curves that fed the FOZ model identification procedure. The second corresponds to the high-level problem, AAD, in the center (reddish area). On this stage, RCHH-LS generated candidate MHs under cardinality ($\varpi$) constraints from a collection of SOs. The result comprised a set of MHs tailored for the low-level problem. Lastly, the comparative evaluation and analysis stage (light blue area) is shown on the right-hand side. At this stage, the tailored MHs were examined to identify the best and worst configurations. The effects of cardinality and hyperparameter tuning were investigated, and the influence of the search operators’ positions was assessed. The performance of these MHs is quantified by the performance metric *Q* (see Eq. (23)), which is the sum of the median and the interquartile range of the objective function value (Eq. (25)); thus, lower values indicate better performance. In addition, the viscoelastic parameters obtained by the selected custom MH were analyzed. Each of these stages will be described in detail below.

### Low-level problem: model identification

**Fig. 4:**
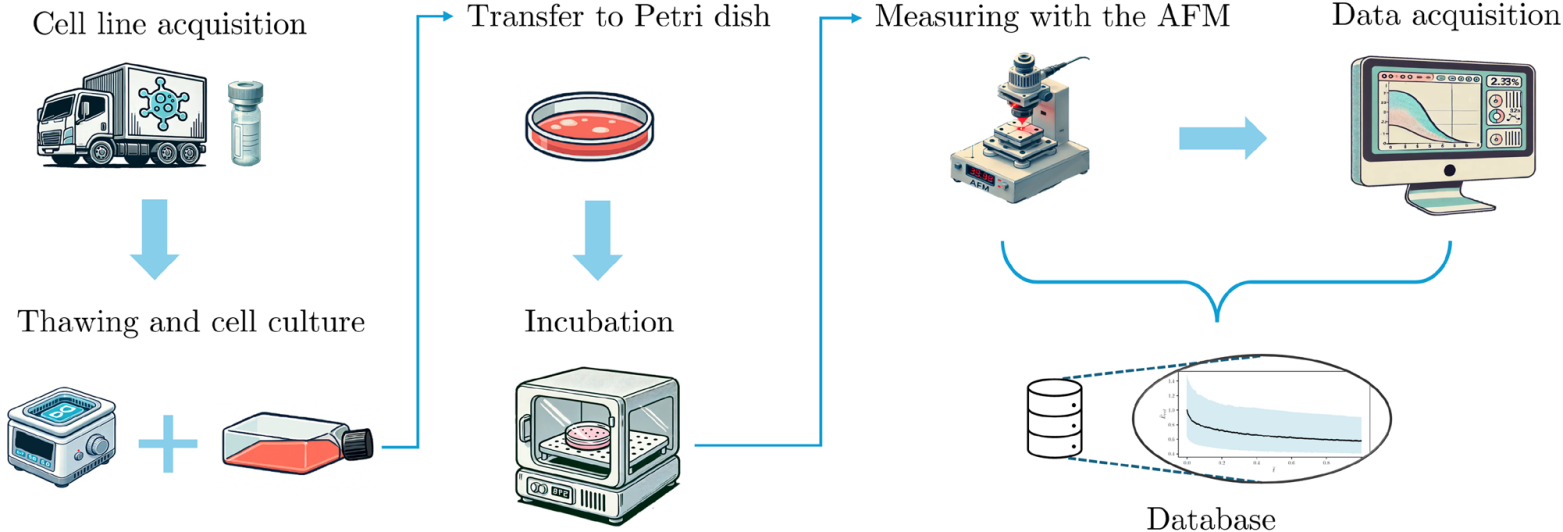
Experimental measurement procedure conducted, from the MG-63 cell line acquisition to the extraction of relaxation curves using Atomic Force Microscopy (AFM).

The low-level problem focuses on characterizing the viscoelastic behavior of human osteoblastic MG-63 cells through force-relaxation curves obtained using AFM and FOZ model. To ensure the reliability of the data, an experimental protocol was followed. First, the process began with acquiring the commercial MG-63 cell line from the American Type Culture Collection (ATCC). The cells were then cultured in 3.5 cm-diameter Petri dishes, using DMEM-F12 medium, supplemented with 10% fetal bovine serum and 1% penicillin-streptomycin (Gibco), and maintained in a humidified incubator containing 5% $\text {CO}_2$ at 37 $^\circ$C, following the guidelines and regulations provided by the ATCC (Manassas, VA, USA). All experimental protocols used in this article were approved by the Comité de Investigación of the Facultad de Ciencias Biológicas of the Universidad Autónoma de Nuevo León. After a 72-hour incubation period, confluence was qualitatively assessed using optical microscopy to ensure adequate coverage and the presence of isolated cells for measurements. Samples were then carefully transferred to the AFM stage for mechanical characterization. At this point, indentation measurements were carried out to obtain the force-relaxation curves. Figure 4 illustrates the complete experimental methodology.

The force curves provided by the AFM were measured on individual cells in their liquid medium. Each cell was indented twice. In the first indentation, the maximum applied load did not exceed 18 nN, and the relaxation was recorded for 5 s. In the second indentation, the load ranged from 18 to 23 nN, with relaxation recorded for 25 s. The loading rate was approximately $2.5\;\mu$m/s. These force ranges were achieved by controlling the extension of the piezoelectric actuator in the approach phase. This produced indentations of 1.73±0.46 $\mu$m for $F<18$ nN and 2.05±0.50 $\mu$m for $F>18$ nN. An SNL-10 cantilever with a conical tip and a nominal spring constant of 0.12 N/m was used for all measurements. Consequently, two distinct groups of force curves were obtained. One comprises data for loads below 18 nN, and the other for loads above 18 nN. Each group contains 21 individual instances. Moreover, the force relaxation curves are fitted by applying a scaling that accounts for the contact geometry between the AFM tip and the cell. The relationship used is^73^,

24 $$\begin{aligned} F(t) = \frac{{C}}{1 - v^2} \, E_{\text {rel}}(t), \end{aligned}$$

where *C* [$\text {m}^2$] depends on the tip geometry. For conical tips, $C = (2/\pi ) \tan (\theta )\delta _0^2$, with $\delta _0$ being the maximum indentation depth. In addition, *v* is the cell’s Poisson ratio, *F* [N] is the load force, $\delta$ [m] is the indentation depth, and $\theta$ ($25^\circ$) is the half-opening angle of the conical tip. Since cells are commonly assumed to be incompressible materials, $v = 0.5$ is used.

Subsequently, the relaxation curves obtained were non-dimensionalized with respect to time *t* [s] and the relaxation modulus $E_{\text {rel}}$ [Pa]. To nondimensionalize time, a scaling factor of 25 s was used for datasets corresponding to forces greater than 18 nN, and 5 s for datasets with forces below that threshold. These values represent the maximum duration of the measurements in each dataset. For the nondimensionalized of $E_{\text {rel}}$, the average of the maximum values across all curves within each dataset was computed. Specifically, a value of $8.1465~\mu$ Pa was used for the high-force group (forces > 18 nN), and $6.1850~\mu$ Pa for the low-force group (forces < 18 nN).

Now, the domain of the low-level problem corresponding to the FOZ model fitting defined in (15) is a four-dimensional space, where the design vector is given by $\bigl (E_{0}, E_{1}, \alpha , \tau \bigr )^\top \in \mathfrak {X}\subseteq \mathbb {R}^{4}$, with $E_0, E_1, \tau \in (0.0, 1.5]$ and $\alpha \in [0.1, 1.0]$. Besides, the objective function is the sum of the Mean Squared Error (MSE) and the Mean Absolute Error (MAE), as shown,

25 $$\begin{aligned} f(E_{0}, E_{1}, \alpha , \tau ) \;=\; \underbrace{\frac{1}{N} \sum _{i=1}^{N} \bigl (\bar{E}_{\textrm{rel}}(\bar{t}_i) \;-\; E_{\textrm{rel}}\bigl (\bar{t}_i; E_0, E_1, \alpha , \tau \bigr )\bigr )^2}_{\text {MSE}} \;+\; \underbrace{\frac{1}{N} \sum _{i=1}^{N} \bigl |\bar{E}_{\textrm{rel}}(\bar{t}_i) \;-\; E_{\textrm{rel}}\bigl (\bar{t}_i; E_0, E_1, \alpha , \tau \bigr )\bigr |}_{\text {MAE}}, \end{aligned}$$

where *N* is the total number of sampling points, $\bar{E}_{\textrm{rel}}(\bar{t}_i)$ represents the experimentally observed relaxation values at time $\bar{t}_i$, and $E_{\textrm{rel}}(\bar{t}_{i}; E_{0}, E_{1}, \alpha , \tau )$ is estimated using the FOZ model. This combination of metrics in the objective function was adopted because the scale and nature of the problem’s error were unknown during the initial implementation. The MSE component increases the sensitivity to large deviations, while the MAE contributes to an evaluation against smaller errors.

### High-level problem: automated algorithm design

The high-level problem focuses on the AAD of custom MHs by combining different SOs selected from a predefined heuristic space. This domain, an essential part of the RCHH-LS process, included a collection of 13 perturbation heuristics, each associated with specific hyperparameters and two selection mechanisms: greedy and direct. To implement this process, we used the CUSTOMHyS framework, freely available at PyPI, which provides a comprehensive library of SOs. CUSTOMHyS is a modular and extensible environment designed to support the development and evaluation of MH algorithms by treating them as configurable building blocks^74^. Table 2 summarizes the SOs used in the design of each candidate MH. During the construction phase, candidate MHs are constructed by selecting combinations of SOs according to a given cardinality $\varpi$, and then refined through a local search stage to improve performance. For this purpose, the HH process was limited to 10 steps, allowing maximum cardinalities of two and three SOs, respectively.

**Table 2:** Collection of search operators utilized to obtain the tailored MH and their parameters variation, hyperparameters, and associated selector.

ID	Perturbator ($h_p$)	Parameters	Hyperparameters	Selector
$h_0$	central_force_dynamic	–	gravity = 0.001, alpha = 0.01, beta = 1.5, dt = 1.0	All
$h_1$	central_force_dynamic	–	gravity = 0.001, alpha = 0.01, beta = 1.5, dt = 1.0	Greedy
$h_2$	differential_mutation	Mutation $\textrm{Scheme}^{b_1}$	num_rands = 1, factor = 1.0	All
$h_3$	differential_mutation	Mutation $\textrm{Scheme}^{b_1}$	num_rands = 1, factor = 1.0	Greedy
$h_4$	differential_mutation	Mutation $\textrm{Scheme}^{b_2}$	num_rands = 1, factor = 1.0	All
$h_5$	differential_mutation	Mutation $\textrm{Scheme}^{b_2}$	num_rands = 1, factor = 1.0	Greedy
$h_6$	firefly_dynamic	$\textrm{Distribution}^{a_2}$	alpha = 1.0, beta = 1.0, gamma = 100.0	All
$h_7$	firefly_dynamic	$\textrm{Distribution}^{a_2}$	alpha = 1.0, beta = 1.0, gamma = 100.0	Greedy
$h_8$	genetic_crossover	$\textrm{PS}^{c_1}$, $\textrm{CM}^{d_3}$	mating_pool_factor = 0.4	All
$h_{9}$	genetic_crossover	$\textrm{PS}^{c_1}$, $\textrm{CM}^{d_3}$	mating_pool_factor = 0.4	Greedy
$h_{10}$	genetic_crossover	$\textrm{PS}^{c_3}$, $\textrm{CM}^{d_3}$	mating_pool_factor = 0.4	All
$h_{11}$	genetic_crossover	$\textrm{PS}^{c_3}$, $\textrm{CM}^{d_3}$	mating_pool_factor = 0.4	Greedy
$h_{12}$	genetic_mutation	$\textrm{Distribution}^{a_2}$	scale = 1.0, elite_rate = 0.1, mutation_rate = 0.25	All
$h_{13}$	genetic_mutation	$\textrm{Distribution}^{a_2}$	scale = 1.0, elite_rate = 0.1, mutation_rate = 0.25	Greedy
$h_{14}$	gravitational_search	–	gravity = 1.0, alpha = 0.02	All
$h_{15}$	gravitational_search	–	gravity = 1.0, alpha = 0.02	Greedy
$h_{16}$	local_random_walk	$\textrm{Distribution}^{a_1}$	probability = 0.75, scale = 1.0	All
$h_{17}$	local_random_walk	$\textrm{Distribution}^{a_1}$	probability = 0.75, scale = 1.0	Greedy
$h_{18}$	random_sample	–	–	All
$h_{19}$	random_sample	–	–	Greedy
$h_{20}$	random_search	$\textrm{Distribution}^{a_2}$	scale = 1.0	Greedy
$h_{21}$	random_search	$\textrm{Distribution}^{a_2}$	scale = 0.01	Greedy
$h_{22}$	spiral_dynamic	–	radius = 0.9, angle = 22.5, sigma = 0.1	All
$h_{23}$	spiral_dynamic	–	radius = 0.9, angle = 22.5, sigma = 0.1	Greedy
$h_{24}$	swarm_dynamic	$\textrm{Distribution}^{a_2}$, $\textrm{SWA}^{e_1}$	factor = 0.7, self_conf = 2.54, swarm_conf = 2.56	All
$h_{25}$	swarm_dynamic	$\textrm{Distribution}^{a_2}$, $\textrm{SWA}^{e_1}$	factor =0.7, self_conf =2.54, swarm_conf =2.56	Greedy
$h_{26}$	swarm_dynamic	$\textrm{Distribution}^{a_2}$, $\textrm{SWA}^{e_2}$	factor =1.0, self_conf =2.54, swarm_conf =2.56	All
$h_{27}$	swarm_dynamic	$\textrm{Distribution}^{a_2}$, $\textrm{SWA}^{e_2}$	factor =1.0, self_conf =2.54, swarm_conf =2.56	Greedy
$h_{28}$	random_flight	$\textrm{Distribution}^{a_3}$	scale = 1.0, beta = 1.5	All
$h_{29}$	random_flight	$\textrm{Distribution}^{a_3}$	scale = 1.0, beta = 1.5	Greedy

Distribution: $^{a_1}$Gaussian, $^{a_2}$Uniform, $^{a_3}$Lévy;  Mutation Scheme: $^{b_1}$current-to-best, $^{b_2}$rand-to-best-and-current

Pairing Scheme (PS): $^{c_1}$Cost, $^{c_3}$Tournament;   Crossover Mechanism (CM): $^{d_3}$Uniform;

Swarm Approach (SWA): $^{e_1}$Inertial, $^{e_2}$Constriction

Each candidate MH has a budget of approximately 10,100 evaluations of the objective function, distributed according to the corresponding cardinality. Specifically, for a cardinality of two operators, 101 iterations were performed with a population of 50 individuals, while for a cardinality of three operators, 70 iterations were conducted with a population of 48 individuals. Furthermore, to evaluate the performance metric defined in (23), each candidate MH was run in 20 independent replications, i.e., solving the low-level problem 20 times. Since the HH process can be computationally expensive, we chose to use the median of the nondimensionalized relaxation curve of the set corresponding to forces greater than 18 nN as a single instance of the low-level problem. As a result of the HH process, the best and worst performing configurations were selected. On the one hand, the best MHs found for each cardinality are denoted as $\text {MH}_*^2$ and $\text {MH}_*^3$. On the other hand, the worst configurations $\text {MH}_{\circledast }^2$ and $\text {MH}_{\circledast }^3$. Additionally, the best performance (Best Perf) and worst performance (Worst Perf) of the selected candidate MHs are provided. The data collected in each replicate are used in the subsequent analysis detailed below.

### Comparative evaluation and analysis

The comparative evaluation and analysis stage was devised to gain a deeper understanding of the AAD, and not to be limited only to the performance of a specific configuration generated using this approach. First, we analyzed how cardinality affects the construction of tailored MHs. Next, we explored the impact of hyperparameter tuning and the influence of the position of search operators within each selected configuration. Finally, we analyzed the viscoelastic parameters obtained by fitting the FOZ model using the best average performing MH. These three steps are detailed in the remainder of this section.

#### Effect of the cardinality of the tailored metaheuristic

To analyze how cardinality (that is, the number of SOs) influences the design of custom MHs, the RCHH-LS model was employed to automatically generate configurations with two and three operators per sequence. For each cardinality value, 20 independent runs of the HH process were carried out. In each of these replicates, the best and the worst sequences obtained were recorded, and the distribution of the performance shown was analyzed. The objective was to evaluate how cardinality affects the performance of the MHs, keeping the number of evaluations of the objective function constant. A map of the performance landscape was also constructed, considering all possible combinations with cardinality two. This allowed us to identify which operators occupy the regions with the best performance to solve the low-level problem.

#### Effect of Hyperparameter Tuning in Metaheuristic Design

In this step, we analyzed the effect of hyperparameter tuning on tailored MHs’ performance. We selected four representative MH configurations from the HH process: two with performances close to the median of the worst-performing sequences, denoted as $\text {MH}_{\circledast }^2$ and $\text {MH}_{\circledast }^3$ for cardinalities two and three; and two with performances close to the median of the best-performing sequences, denoted as $\text {MH}_{*}^2$ and $\text {MH}_{*}^3$. Each selected MH was subjected to a hyperparameter tuning process using the Optuna framework^46^. Parameter search spaces were defined based on the ranges associated with each operator, as shown in Table 4.

For each MH and parameters configuration, 20 replications were executed to ensure robustness and evaluate the results’ variability. Furthermore, we evaluated the performance of each tuned MH in all 21 available instances (relaxation curves for forces greater than 18 nN) to examine their generalization capacity. This experimental procedure permitted us to assess how hyperparameter tuning can improve a given MH’s performance and determine whether specific configurations can reach or surpass the effectiveness of those configurations obtained through the HH process alone.

#### Effect of the position of search operators on metaheuristic performance

In this step, we selected a base MH of cardinality three to evaluate the impact of SOs’ arrangement and position on MHs’ performance. Five additional MHs were derived from this configuration by applying specific modifications: three were obtained by removing one SO, and two by exchanging selected SOs’ positions. Each variant of MH was run on all 21 instances of the problem (relaxation curves with a strength greater than 18 nN), with 20 runs per instance. The performance values obtained from these runs were analyzed and visualized per configuration using box plots. For each configuration, we showed the distribution of cumulative performance across instances and the average performance of each run over the 21 instances.

#### Analysis of viscoelastic parameters

Two independent data sets were analyzed to evaluate the performance of the fitting FOZ model and the selected $\text {MH}_*^{3}$. These data sets, not used during the HH process, consist of relaxation curves obtained by AFM on MG-63 cells, sorted by strength regime: $F<18$ nN and $F>18$ nN. Each data set includes 21 instances, each replicated 30 times to ensure statistical reliability. The curves were fitted using $\text {MH}_*^{3}$, and seven error metrics were calculated to assess the model accuracy: MSE, Root MSE (RMSE), MAE, Maximum Absolute Error (Max Error), Coefficient of Determination ($R^{2}$), Normalized RMSE (NRMSE), and Bias. Statistical indicators for each condition, such as median, interquartile range, coefficient of variation, and variance, were calculated. Besides, a nonparametric Mann-Whitney *U* test was performed to assess whether the distributions of the parameters obtained differed significantly between the two strength conditions.

## Experimental results

This section presents and analyzes the experimental results obtained following the methodology described in Section “Methodology”. In addition, to carry out the experimental evaluation, all implementations were developed in Python v3.9 and executed in a controlled computational environment. Specifically, we utilized a virtualized Windows 11 Pro system on an Intel(R) Xeon(R) Gold 5218 CPU at 2.30 GHz, with 16 allocated logical processors and 32 GB of RAM. The results obtained in each experimental phase are shown below.

### Effect of the cardinality of the tailored metaheuristic

**Fig. 5:**
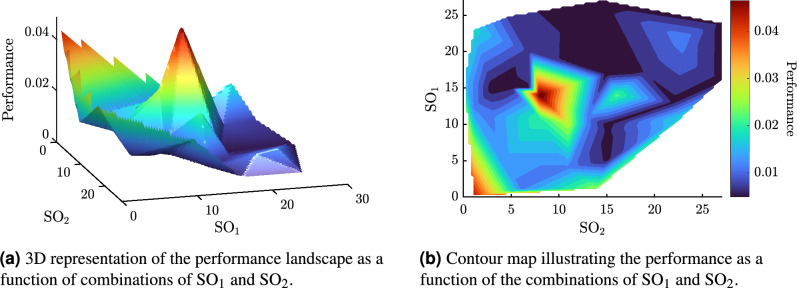
3D Performance landscape and contour map for two-operator metaheuristics in the hyper-heuristic process. Performance is quantified by the performance metric *Q* (see Eq. (23)), which is the sum of the median and the interquartile range of the objective function value; thus, lower values indicate better performance.

This section evaluates how the cardinality of a tailored MH, defined as the number of SOs per configuration, affects its performance in solving the low-level problem. Table 3 presents the results obtained during the 20 runs of the HH process, assessed for cardinalities two and three. In each replicate, the sequences or custom MHs that achieved the best and worst performance were identified, along with their corresponding performance. For cardinality two, the sequence with the best performance was $\text {MH} = [h_{15},h_{27}]$, achieving a performance of $4.89 \times 10^{-3}$, while the sequence with the worst performance was $\text {MH} = [h_{8},h_{14}]$, with a performance of $4.99 \times 10^{-2}$. The best MH sequences with cardinality two presented an average and a standard deviation performance of $6.16 \times 10^{-3}\pm 0.8 \times 10^{-3}$, in contrast to the worst sequences, which obtained $2.09 \times 10^{-2}\pm 1.14 \times 10^{-2}$. In addition, a linear interpolation was conducted from the results to analyze the performance landscape of the low-level problem solved with an MH with cardinality two (see Fig. 5). This result reveals that combinations of operators, such as swarm dynamics and gravitational search, were associated with the best-performing regions. This behavior depends on the sequence of operators and the position in which they are selected. First, the dynamic swarm perturbator modulates the velocity of the particles, leading the population to promising regions while preserving diversity, thus providing directed exploration. Immediately thereafter, the gravitational search operator applies a global attraction, clustering the swarm within the already localized minima and providing controlled exploitation. This ordered synergy between velocity-controlled exploration and attraction-based refinement has proven advantageous in continuous multimodal problems^75,76^. However, it is worth remembering that no algorithm is universally optimal; rather, its success depends on how well its search dynamics are matched to the problem at hand, as the no-free-lunch theorem tells us. The present results suggest that our low-level task requires an MH capable of combining broad exploration with fine-grained exploitation first to discover promising regions and then to refine the solution without sacrificing population diversity. If diversity is lost too early, the search risks being trapped in suboptimal neighborhoods; conversely, if exploitation is too weak, convergence may be slow or erratic. Thus, a balanced combination of SOs is crucial to achieve the desired returns. In contrast, combinations of genetic crossover with gravitational search and random samples dominated the least-performing regions. Figure 5 clearly indicates that there are combinations that help improve performance and others that do not. This is due to the way the search process is initiated, by having a gravitational search type operator it may very quickly drive the population to a local minimum, and then to try to refine those solutions the genetic crossover operator comes in, which will try to explore but will depend on what was done by the previous perturbator. Premature exploitation does not allow this problem to achieve the best results.

**Table 3:** Summary of the best and worst-performing MH sequences across 20 runs of the HH process for cardinalities two and three. The table includes the sequences (Seq) and their corresponding performance (Perf).

Rep	Cardinality 3				Cardinality 2			
	Best		Worst		Best		Worst	
	Seq	Perf	Seq	Perf	Seq	Perf	Seq	Perf
1	$[h_3, h_{14}, h_8]$	0.0065	$[h_1, h_{2}, h_6]$	0.0114	$[h_{15}, h_{10}]$	0.00571	$[h_{11}, h_{10}]$	0.0276
2	$[h_{26}, h_{25}, h_{27}]$	0.0030	$[h_{16}, h_{1}, h_{17}]$	0.0121	$[h_{15}, h_{17}]$	0.00525	$[h_{4}, h_{5}]$	0.0139
3	$[h_{26}, h_{16}, h_{5}]$	0.0054	$[h_{14}, h_{24}, h_{21}]$	0.0108	$[h_{7}, h_{20}]$	0.00800	$[h_{16}, h_{14}]$	0.0227
4	$[h_{3}, h_{9}, h_{23}]$	0.0046	$[h_{0}, h_{6}, h_{16}]$	0.0112	$[h_{14}, h_{26}]$	0.0057	$[h_{8}, h_{14}]$	0.0499
5	$[h_{15}, h_{12}, h_{11}]$	0.0029	$[h_{23}, h_{14}, h_{9}]$	0.0222	$[h_{15}, h_{5}]$	0.0064	$[h_{1}, h_{0}]$	0.0455
6	$[h_{23}, h_{16}, h_{15}]$	0.0061	$[h_{1}, h_{4}, h_{9}]$	0.0119	$[h_{15}, h_{27}]$	0.0049	$[h_{1}, h_{12}]$	0.0133
7	$[h_{19}, h_{15}, h_{11}]$	0.0050	$[h_{14}, h_{15}, h_{16}]$	0.0372	$[h_{15}, h_{27}]$	0.0053	$[h_{8}, h_{11}]$	0.0215
8	$[h_{19}, h_{17}, h_{3}]$	0.0058	$[h_{17}, h_{21}, h_{14}]$	0.0272	$[h_{15}, h_{9}]$	0.0064	$[h_{1}, h_{0}]$	0.0381
9	$[h_{3}, h_{1}, h_{15}]$	0.0029	$[h_{17}, h_{21}, h_{2}]$	0.0118	$[h_{22}, h_{13}]$	0.0062	$[h_{23}, h_{22}]$	0.0124
10	$[h_{5}, h_{0}, h_{9}]$	0.0029	$[h_{17}, h_{1}, h_{14}]$	0.0288	$[h_{9}, h_{17}]$	0.0070	$[h_{23}, h_{11}]$	0.0171
11	$[h_{14}, h_{26}, h_{5}]$	0.0030	$[h_{0}, h_{12}, h_{18}]$	0.0114	$[h_{7}, h_{15}]$	0.0069	$[h_{0}, h_{16}]$	0.0185
12	$[h_{15}, h_{27}, h_{5}]$	0.0029	$[h_{1}, h_{4}, h_{21}]$	0.0117	$[h_{3}, h_{15}]$	0.0052	$[h_{6}, h_{1}]$	0.0128
13	$[h_{27}, h_{26}, h_{3}]$	0.0029	$[h_{16}, h_{1}, h_{0}]$	0.0149	$[h_{27}, h_{24}]$	0.0061	$[h_{1}, h_{23}]$	0.0189
14	$[h_{26}, h_{5}, h_{23}]$	0.0029	$[h_{10}, h_{8}, h_{5}]$	0.0125	$[h_{27}, h_{16}]$	0.0060	$[h_{11}, h_{4}]$	0.0127
15	$[h_{17}, h_{22}, h_{16}]$	0.00600	$[h_{14}, h_{13}, h_{21}]$	0.0213	$[h_{5}, h_{15}]$	0.0065	$[h_{14}, h_{1}]$	0.0261
16	$[h_{14}, h_{10}, h_{3}]$	0.0030	$[h_{9}, h_{8}, h_{0}]$	0.0126	$[h_{5}, h_{25}]$	0.0076	$[h_{3}, h_{11}]$	0.0127
17	$[h_{16}, h_{1}, h_{11}]$	0.0044	$[h_{16}, h_{17}, h_{12}]$	0.0105	$[h_{15}, h_{8}]$	0.0059	$[h_{23}, h_{19}]$	0.0107
18	$[h_{15}, h_{26}, h_{1}]$	0.0029	$[h_{4}, h_{20}, h_{17}]$	0.0106	$[h_{19}, h_{17}]$	0.0074	$[h_{13}, h_{11}]$	0.0121
19	$[h_{22}, h_{10}, h_{14}]$	0.0033	$[h_{4}, h_{20}, h_{2}]$	0.0103	$[h_{14}, h_{27}]$	0.0055	$[h_{1}, h_{23}]$	0.0189
20	$[h_{26}, h_{23}, h_{0}]$	0.0058	$[h_{3}, h_{4}, h_{15}]$	0.0103	$[h_{3}, h_{15}]$	0.0054	$[h_{13}, h_{10}]$	0.0121

**Fig. 6:**
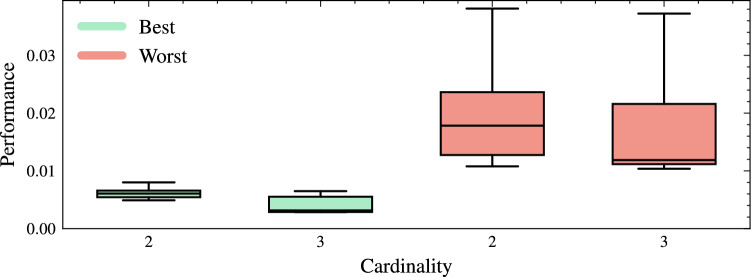
Performance comparison between the best and worst MH sequences for cardinalities two and three, obtained from the hyper-heuristic process with 20 runs. Performance is calculated as the median plus the inter-quartile range of the objective-function values across replications; lower values (approaching zero) denote better results..

Figure 6 compares the performance distributions of the best and worst MHs at cardinalities two and three obtained in the HH process in 20 runs. For cardinality two, the best-performing sequences achieved an average performance that outperformed the worst-performing sequences by approximately 70.7%, demonstrating the significant advantage of selecting optimal operator combinations such as $\text {MH} = [h_{15}, h_{27}]$ over less efficient ones such as $\text {MH} = [h_8, h_{14}]$. For cardinality three, the best-performing sequences exhibited an even more significant improvement, outperforming the worst-performing sequences by 75.3%. Interestingly, the worst-performing sequences for cardinalities two and three presented similar performance distributions, with a slight difference in their medians. It is observed that poorly selected operator combinations produce suboptimal results, regardless of cardinality. When comparing the best sequences of cardinalities two and three, we notice that three operators offered a performance advantage of approximately 50.6% over the best two-operator sequences. Among the top-performing combinations for cardinality three, the operators swarm dynamic, gravitational search, and differential mutation stand out.

### Effect of hyperparameter tuning in metaheuristic design

This section details how hyperparameter tuning affects the performance of automatically designed MHs. We evaluated how it improves the performance of the best MHs in the HH process and investigated whether the worst solutions can achieve competitive performances. First, MHs with configurations close to the median performance obtained by the worst sequences in the HH process were selected. In the case of cardinality two, the sequence $\text {MH}_{\circledast }^2 = [h_0, h_{16}]$ (replicate 11) was identified, which presented an initial performance of $1.85 \times 10^{-2}$. Besides, for cardinality three, the MH comprising the sequence $\text {MH}_{\circledast }^3 = [h_{23}, h_{14}, h_9]$ (replicate 6) was selected, with an initial performance of $1.19 \times 10^{-2}$.

**Fig. 7:**
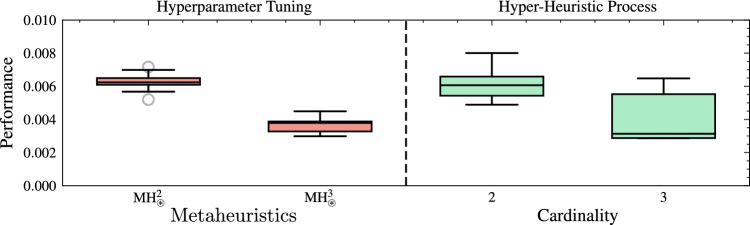
Performance comparison of metaheuristics tuned via Optuna and those designed via the hyper-heuristic process, evaluated for cardinalities two and three in 20 runs. Where $\text {MH}_{\circledast }^2=[h_0,h_{16}]$ and $\text {MH}_{\circledast }^3=[h_{23},h_{14},h_{9}]$ are the worst performing metaheuristics of cardinality 2 and 3 selected from the hyper-heuristic process and with adjustment of their hyperparameters. The performance is calculated by (23)..

Figure 7 depicts the performance comparison between these selected MHs, tuned using the Optuna framework, and those designed through the HH process, evaluated for both cardinalities. For cardinality two, the median value achieved by the tuned MH ($\text {MH}_{\circledast }^2$) was $6.2 \times 10^{-3}$, which represents an improvement of 66.3% over the initial performance obtained in the HH process. Moreover, this tuned performance is comparable to the best MHs designed using the HH process, whose median is $6.1 \times 10^{-3}$. In the case of cardinality three, the tuned $\text {MH}_{\circledast }^3$ achieved a median performance of $3.8 \times 10^{-3}$, achieving a significant improvement of 78.8% over its initial performance. This performance is also very close to that of the best MHs in the HH process for cardinality three, with a median of $3.1 \times 10^{-3}$.

Correct hyperparameter tuning can transform initially unfavorable configurations into highly competitive solvers, achieving performances that approach, and in some cases even equal, those of the best MHs obtained through the HH process. However, an interesting discussion arises when evaluating the performance of the best configurations of cardinality two and three during tuning their hyperparameters. To explore this, we also selected the best two- and three-operator MHs that closely align with the median performance achieved by the HH process. For cardinality two, the chosen MH corresponds to $\text {MH}_{*}^2 = [h_{27}, h_{24}]$ (replicate 13), achieving a performance of $6.1 \times 10^{-3}$, very close to the median performance of the HH process for cardinality two ($6.05\times 10^{-3}$). Similarly, for cardinality three, the selected MH is $\text {MH}_{*}^3 = [h_{26}, h_{25}, h_{17}]$ (replicate 2), with a performance of $3.0 \times 10^{-3}$, also aligning closely with the median of $3.13 \times 10^{-3}$. Table 4 presents the configurations of the selected MHs ($\text {MH}_{\circledast }^2$ and $\text {MH}_{\circledast }^3$), highlighting the hyperparameters identified for tuning.

**Table 4:** Best and worst metaheuristics configuration generated by the hyper-heuristics method with cardinality two and three.

MHs	SOs	SHs	Parameters	hyperparameters
$\text {MH}_{\circledast }^2$	$h_0$	$h_p=$ Central force dynamic $h_s=$ Direct	–	gravity, alpha $\in (0, 0.01]$, beta $\in (1, 2)$
	$h_{16}$	$h_p=$ Local random walk $h_s=$ Direct	–	scale $\in (0,1]$, probability $\in (0,1]$
$\text {MH}_{\circledast }^3$	$h_{23}$	$h_p=$ Spiral dynamic $h_s=$ Greedy	–	radius $\in (0,1)$, angle $\in (0^\circ , 360^\circ )$, sigma $\in [0,1)$
	$h_{14}$	$h_p=$ Gravitational search $h_s=$ Direct	–	gravity $\in (0,1]$, alpha $\in (0,0.1]$
	$h_{9}$	$h_p=$ Genetic crossover $h_s=$ Greedy	PS = Roulette wheel pairing CM = Uniform	mating_pool_factor $\in [0,1]$
$\text {MH}_{*}^2$	$h_{27}$	$h_p=$ Swarm dynamic $h_s=$ Greedy	SWA = Constrained	factor $\in (0,1)$, self_conf , swarm_conf $\in (0,4]$
	$h_{24}$	$h_p=$ Swarm dynamic $h_s=$ Direct	SWA = Inertial	factor $\in (0,1)$, self_conf , swarm_conf $\in (0,4]$
$\text {MH}_{*}^3$	$h_{26}$	$h_p=$ Swarm dynamic $h_s=$ Direct	SWA = Constrained	factor $\in (0,1)$, self_conf , swarm_conf $\in (0,4]$
	$h_{25}$	$h_p=$ Swarm dynamic $h_s=$ Greedy	SWA = Inertial	factor $\in (0,1)$, self_conf , swarm_conf $\in (0,4]$
	$h_{17}$	$h_p=$ Local random walk $h_s=$ Greedy	–	scale $\in (0,1]$, probability $\in (0,1]$

SWA: swarm_approach,    PS: pairing_scheme,    CM: crossover_mechanism

**Fig. 8:**
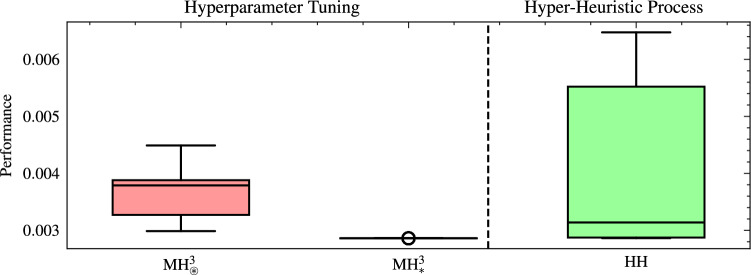
Performance comparison between metaheuristics adjusted via hyperparameter tuning ($\text {MH}_{*}^3 =[h_{26},h_{25},h_{17}]$ and $\text {MH}_{\circledast }^3 =[h_{23},h_{14},h_{9}]$) and those generated by the hyper-heuristic process across 20 runs. Performance is calculated as the median plus the inter-quartile range of the objective-function values across replications; lower values (approaching zero) denote better results..

Figure 8 highlights the performance of the MH with three operators when subjected to hyperparameter fine-tuning, reaching an average mark of $2.86 \times 10^{-3}$ with a standard deviation of $2.43 \times 10^{-7}$. This result not only surpasses the performance obtained from the HH process but also exhibits high repeatability. Note that this is not intended to detract from the HH process’s ability to generate MHs with competitive performance. In contrast, these results confirm that combining both approaches can produce solvers with outstanding performance. To determine whether the tuned $\text {MH}_{*}^{3}$ outperforms the worst configuration of the same cardinality $\text {MH}_{\circledast }^{3}$, we applied a one-sided Mann-Whitney U test to the fitness values obtained in 20 independent replications per MH. The null hypothesis stated that $\text {MH}_{*}^{3}$ offers no performance advantage over $\text {MH}_{\circledast }^{3}$, whereas the alternative posited a clear superiority of $\text {MH}_{*}^{3}$. The test yielded a one-tailed *p*-value of $3.36 \times 10^{-8}$, and a rank-biserial correlation of 0.99; thus, the null hypothesis is rejected, confirming that $\text {MH}_{*}^{3}$ attains significantly better fitness than $\text {MH}_{\circledast }^{3}$ and validating its superior performance.

**Fig. 9:**
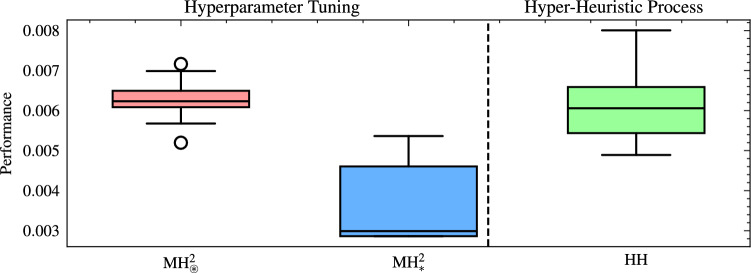
Performance comparison between metaheuristics adjusted via hyperparameter tuning ($\text {MH}_{*}^2 =[h_{27},h_{24}]$ and $\text {MH}_{\circledast }^2 =[h_{0},h_{16}]$) and those generated by the hyper-heuristic process across 20 runs. The performance is calculated by (23).

As in the previous analysis, we utilized the Optuna framework to evaluate the quality of $\text {MH}_{\circledast }^2$ and $\text {MH}_{*}^2$ over 20 runs. Figure 9 presents the performance comparison between $\text {MH}_{*}^2$ and $\text {MH}_{\circledast }^2$, and the overall HH process. In the case of $\text {MH}_{*}^2$, we notice a performance of $2.86 \times 10^{-3}\pm 2.5 \times 10^{-4}$, reflecting remarkable stability and consistency across replicates. Compared to its initial median performance ($6.09 \times 10^{-3}$), the improvement reached 53.1%, revealing the significance of including a hyperparameter tuning stage in the automated design of metaheuristics. In addition, $\text {MH}_{\circledast }^2$ presented a performance of $6.13 \times 10^{-3}\pm 4.8 \times 10^{-4}$, which shows a lower performance than $\text {MH}_{*}^2$. However, there was an improvement of 64.3% over its initial results. Therefore, while hyperparameter tuning enhances even the less-promising setups, selecting the right SOs is critical. To verify that the most promising tuned MH of cardinality two ($\text {MH}_{*}^{2}$) outperforms the worst configuration of the same cardinality ($\text {MH}_{\circledast }^{2}$), we applied a one-sided Mann-Whitney U test to the fitness scores obtained in 20 independent replications per algorithm. The null hypothesis stated that $\text {MH}_{*}^{2}$ offers no performance advantage over $\text {MH}_{\circledast }^{2}$, whereas the alternative posited a clear superiority of $\text {MH}_{*}^{2}$. The test yielded a one-tailed *p*-value of $3.95 \times 10^{-8}$, and a rank-biserial correlation of 0.995; thus, the null hypothesis is rejected at the 0.05 level, confirming that $\text {MH}_{*}^{2}$ achieves significantly better fitness than $\text {MH}_{\circledast }^{2}$ and validating its superior performance. Not all configurations can achieve competitive performance solely through tuning, emphasizing the importance of carefully designing initial operator combinations in the automated design process.

**Fig. 10:**
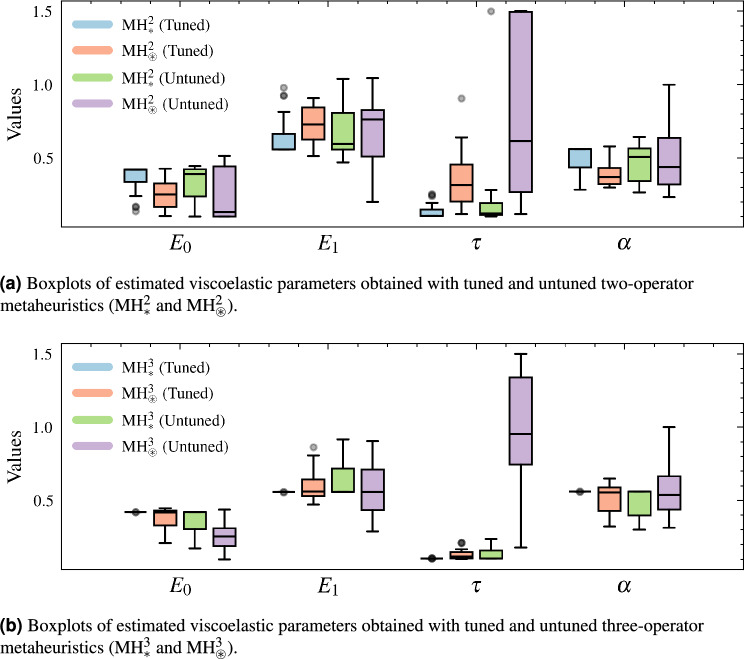
Comparison of estimated viscoelastic parameters ($E_0$, $E_1$, $\tau$, and $\alpha$) across tuned and untuned metaheuristics. Each boxplot summarizes the variability and median of the parameters obtained from 20 different runs of each MH.

Thus far, we have shown that each MH configuration delivers a different overall performance depending on its SOs configuration and subsequent tuning. Figure 10 illustrates that these performance differences directly translate into the stability of the FOZ parameters. The three-operator tuned configuration, ${\mathrm {MH}}_{*}^{3}$ (adjusted), produces an $E_{0}$ value of 0.420 with a coefficient of variation (CV) of just 0.043%. In contrast, the worst two-operator untuned configuration, ${\mathrm {MH}}_{\circledast }^{2}$ (unadjusted), yields 0.407 with a CV of 73.6%, representing a 99.94% reduction in dispersion. A similar pattern is observed for $E_{1}$: ${\mathrm {MH}}_{*}^{3}$ (adjusted) achieves 0.558 with a CV of 0.12%, while ${\mathrm {MH}}_{\circledast }^{2}$ (unadjusted) provides 0.763 with a CV of 37.7%, corresponding to a 99.68% decrease in variability. The most sensitive parameter, $\tau$, drops from 0.616 s with a CV of 71.9% in ${\mathrm {MH}}_{\circledast }^{2}$ (unadjusted) to 0.105 s with only 0.55% CV in ${\mathrm {MH}}_{*}^{3}$ (adjusted), reducing dispersion by 99.23%. Finally, the fractional exponent $\alpha$ converges to 0.560 with a CV of 0.065% in ${\mathrm {MH}}_{*}^{3}$ (adjusted), whereas ${\mathrm {MH}}_{\circledast }^{2}$ (unadjusted) exhibits 0.439 with 46.4% CV, amounting to a 99.86% reduction. These quantitative differences confirm that adding a third operator and applying automated tuning not only improve global fitness, but also yield reproducible estimates of $E_{0}$, $E_{1}$, $\tau$, and $\alpha$.

**Fig. 11:**
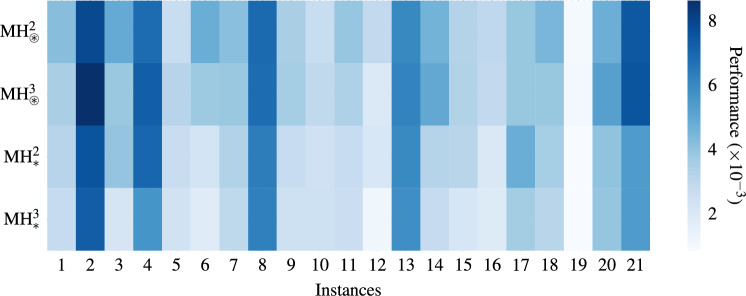
Performance heat map of four metaheuristics in 21 instances. For each instance, 20 replicates were performed to obtain the performance using (23).

Furthermore, we evaluated each MH’s performance individually on all 21 available instances to get a broad perspective. Recall that in previous analyses, we addressed the low-level problem by using the median behavior of all available instances as the curve to be tuned. Table 5 details each MH’s hyperparameters and displays the configuration that best approximates the median performance after 20 runs. In this context, Fig. 11 displays the performance of each MH in different instances. We solve each instance using 20 different replicates and calculate the performance using (23). $\text {MH}_{*}^3$ exhibits the best overall performance, that is, it renders a mean of $3.307\times 10^{-3}$ and dominates in 19 of 21 instances. In the second place is $\text {MH}_{*}^2$, with an overall mean of $3.762\times 10^{-3}$ and two instances in which it obtains the best performance. Nonetheless, $\text {MH}_{{\circledast }}^2$ and $\text {MH}_{\circledast }^3$ report higher means with values of $4.439\times 10^{-3}$ and $4.348\times 10^{-3}$, respectively. Notably, all MHs obtain favorable results in instance 19, while instance 2 is the worst evaluated.

**Table 5:** Selected Hyperparameters for cardinality two and three MHs.

MHs	SOs	Selected hyperparameters
$\text {MH}_{\circledast }^2$	$h_0$	gravity $= 0.021$, alpha $=0.0684$, beta $=1.667$
	$h_{16}$	scale $=0.899$, probability $= 0.9507$
$\text {MH}_{\circledast }^3$	$h_{23}$	radius $=0.234$, angle $=163.17^{\circ }$, sigma $=0.838$
	$h_{14}$	gravity $=0.919$, alpha $=0.0847$
	$h_{9}$	mating_pool_factor $=0.7$
$\text {MH}_{*}^2$	$h_{27}$	factor $=0.197$, self_conf $=1.386$, swarm_conf $=2.055$
	$h_{24}$	factor $=0.734$, self_conf $=0.607$, swarm_conf $=2.092$
$\text {MH}_{*}^3$	$h_{26}$	factor $=0.854$, self_conf $=2.317$, swarm_conf $=2.753$
	$h_{25}$	factor $=0.779$, self_conf $=3.359$, swarm_conf $=3.834$
	$h_{17}$	scale $=0.695$, probability $=0.681$

### Effect of the position of search operators on metaheuristic performance

**Fig. 12:**
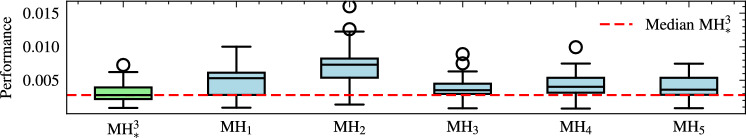
Cumulative performance distribution of the metaheuristic $\text {MH}_*^3$ and its variations across instances. The resulting configurations from operator elimination or reordering are shown, with the median of $\text {MH}_*^3$ indicated as a reference using a red dotted line. Specifically, $\text {MH}_1 = [h_{25}, h_{17}]$, $\text {MH}_2 = [h_{26}, h_{17}]$, $\text {MH}_3 = [h_{26}, h_{25}]$, $\text {MH}_4 = [h_{17}, h_{25}, h_{26}]$, and $\text {MH}_5 = [h_{26}, h_{17}, h_{25}]$.

To better understand the importance of SOs in tailored MHs, we analyze the impact of their variations on performance by eliminating or reordering them according to their position in the sequence. For this purpose, we take as reference the MH constructed and adjusted for cardinality three, denoted $\text {MH}_*^3$. Recall that $\text {MH}_*^3$ comprises the following search operators: $\text {MH}_*^3=[h_{26}, h_{25}, h_{17}]$. Figure 12 presents the cumulative performance distribution for $\text {MH}_*^3$ and its variants. Each boxplot represents a specific configuration derived from $\text {MH}_*^3$. In particular, $\text {MH}_1$, $\text {MH}_2$, and $\text {MH}_3$ correspond to the elimination of the first, second, and third operators. In addition, $\text {MH}_4$ and $\text {MH}_5$ represent configurations in which operator one is exchanged for operator three and operator two for operator three.

A closer examination of the average performance across all instances reveals that removing the second SO ($h_{25}$), that is, $\text {MH}_2=[h_{26}, h_{17}]$, yields the worst average performance, with a value of $7.319\times 10^{-3}$, which is equivalent to a degradation of 160% over $\text {MH}_*^3$, whose value is $2.806\times 10^{-3}$. Therefore, this operator serves as an essential element within the $\text {MH}_*^3$ setup. Similarly, deleting the first SO ($h_{26}$), resulting in $\text {MH}_1 = [h_{25}, h_{17}]$, increases the performance metric to $5.306\times 10^{-3}$, which represents 89% more than the performance of $\text {MH}_*^3$. Furthermore, removing the third SO ($\text {MH}_3 = [h_{26}, h_{25}]$) leads to 25% degradation, with a performance value of $3.522\times 10^{-3}$, indicating that it is the least critical SO in the obtained configuration for $\text {MH}_*^3$. Lastly, $\text {MH}_4=[h_{17}, h_{25}, h_{26}]$ and $\text {MH}_5=[h_{26}, h_{17}, h_{25}]$ are the least affected in terms of performance, however, performance will be affected compared to the initial configuration ($\text {MH}_*^3$).

In general, these results demonstrate the degree of importance of $h_{25}$ and $h_{26}$ in the success of $\text {MH}_*^3$, as well as the significance of their position, indicating that operator combinations are not commutative.

In particular, the $\text {MH}_*^3$ integrates the $h_{26}$, $h_{25}$ and $h_{17}$ operators (see Table 4). The first two belong to the swarm dynamics family, implemented in complementary variants, and the third corresponds to a local random walk. This combination leverages the individual capabilities of each perturbator to strike a balance between exploring and exploiting the search space. The operator $h_{26}$ with a constrained variant introduces a constriction factor that limits the magnitude of the swarm velocity^75,77^, rapidly concentrating the population in low-error basins without overshooting them; next, $h_{25}$ with an inertial variant, reinjects an inertial weight to the velocity, preserving diversity and favoring the avoidance of local minima^75,77^; finally, $h_{17}$ extends the exploration achieved by $h_{25}$ through its local random walk, refining the search around the best solutions. The performance shown by the $\text {MH}_*^3$ sequence reflects the nature of the low-level problem, and by highlighting the nonlinearity caused by the Mittag-Leffler function dependent on the value of $\alpha$, it generates local minima and ridges that require a delicate balance between global exploration and fine exploitation.

### Analysis of viscoelastic parameters

Two data sets were analyzed to evaluate the performance of the FOZ model and the tailored MH ($\text {MH}_*^3$) obtained and fitted. These data sets comprise relaxation curves obtained from AFM measurements, classified by the applied force as greater than 18 nN and less than 18 nN. Each data set consists of 21 instances, and each instance was replicated 30 times. Different metrics were calculated for each force condition to ensure the model’s robustness and correct fit with the experimental data.

Table 6 shows that the model achieved high accuracy for forces exceeding 18 nN, reflected in a median MSE of $1.62\times 10^ {-5}$ and a median $R^2$ of 0.9976. Similarly, performance remained robust for forces below 18 nN, with a slightly lower MSE and comparable $R^2$. The Coefficient of Variation (CV) for the $R^2$ metric remained below 0.01 in both cases, indicating strong stability in the model’s explanatory capacity.

A closer inspection of the dispersion metrics reveals that the error variability decreases appreciably when the load exceeds 18 nN. Specifically, the coefficients of variation drop from 0.7758 to 0.6110 for RMSE, from 0.8005 to 0.6225 for MAE, and from 0.7848 to 0.6455 for NRMSE. The stability of the coefficient of determination also increases, that is, the CV of $R^{2}$ is halved (0.010 to 0.005) and its IQR contracts by 34%. Taken together, these reductions indicate a substantially tighter spread of residual errors in the greater than 18 nN regime, making this loading condition preferable when seeking to maximize robustness and repeatability.

**Table 6:** Statistical indicators for each error metric computed across 21 instances with 30 replications per instance, divided by force condition (greater or less than 18 nN).

Force	Statistic	MSE	RMSE	MAE	Max Error	${R^2}$	NRMSE	Bias
$>18$ nN	Median	$1.62 \times 10^{-5}$	$4.04 \times 10^{-3}$	$2.77 \times 10^{-3}$	$3.43 \times 10^{-2}$	0.9976	$7.12 \times 10^{-3}$	$8.52 \times 10^{-5}$
	IQR	$4.21 \times 10^{-5}$	$4.08 \times 10^{-3}$	$2.32 \times 10^{-3}$	$4.51 \times 10^{-2}$	$5.02 \times 10^{-3}$	$5.14 \times 10^{-3}$	$4.19 \times 10^{-4}$
	CV	0.9773	0.6110	0.6225	1.0494	0.0050	0.6455	8.7224
	Variance	$8.06 \times 10^{-10}$	$6.077 \times 10^{-6}$	$3.00 \times 10^{-6}$	$1.29 \times 10^{-3}$	$2.52 \times 10^{-5}$	$2.11 \times 10^{-5}$	$5.52 \times 10^{-7}$
$<18$ nN	Median	$1.23 \times 10^{-5}$	$3.51 \times 10^{-3}$	$2.64 \times 10^{-3}$	$2.41 \times 10^{-2}$	0.9970	$1.02 \times 10^{-2}$	$-8.01 \times 10^{-5}$
	IQR	$1.46 \times 10^{-5}$	$2.01 \times 10^{-3}$	$1.64 \times 10^{-3}$	$3.54 \times 10^{-2}$	$7.59 \times 10^{-3}$	$8.91 \times 10^{-3}$	$2.44 \times 10^{-4}$
	CV	1.4891	0.7758	0.8005	1.609	0.010	0.7848	$-6.4844$
	Variance	$2.69 \times 10^{-9}$	$7.42 \times 10^{-6}$	$4.46 \times 10^{-6}$	$1.51 \times 10^{-3}$	$1.16 \times 10^{-4}$	$6.44 \times 10^{-5}$	$2.69 \times 10^{-7}$

The metrics are: MSE (Mean Squared Error), RMSE (Root Mean Squared Error), MAE (Mean Absolute Error), Max Error (Maximum Absolute Error), $R^2$ (Coefficient of Determination), NRMSE (Normalized RMSE), and Bias.

After verifying that $\text {MH}_*^3$ approach produces highly accurate and consistent performance across different instances, we examine the parameters obtained that solve the low-level optimization problem. The FOZ model comprises four parameters to characterize cell viscoelasticity: $E_0$, $E_1$, $\tau$, and $\alpha$. The first two parameters account for the cells’ elastic part, while the latter two represent their viscous behavior. As a viscoelastic material, the cell exhibits properties that vary with experimental conditions, such as loading rate and applied force^78^. For example, when measuring the Young modulus of a cell, this value increases directly with increasing force. To evaluate how these parameters behave under different conditions, we performed a comparative statistical analysis by dividing the data set into two groups based on the applied load.

**Fig. 13:**
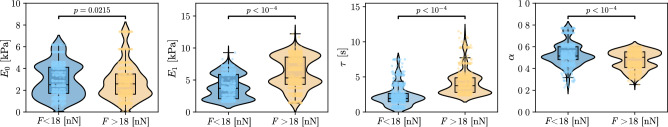
Distribution of the fractional-order Zener model parameters ($E_0$, $E_1$, $\tau$, and $\alpha$) obtained from the fitted relaxation curves under two force regimes: $F < 18$ nN and $F > 18$ nN. Each violin plot displays the median, interquartile range, and distribution of parameter values across 21 instances, each with 30 replications. The statistical significance was assessed using the Mann-Whitney U test, with corresponding *p*-values indicated above each comparison.

Figure 13 illustrates the statistical distributions of the four parameters of the FOZ model under two distinct mechanical load conditions. The violin plots combine kernel density estimation with embedded boxplots, enabling a comprehensive visualization of each parameter’s central tendency and variability. The parameter $E_0$ denotes the residual cell stiffness at long times, i.e., it reflects the elastic resistance after the cell has reached mechanical equilibrium in response to the applied force. The smaller values of $E_0$ correspond to more deformable cells since less resistance remains once the initial deformation has stabilized.

In addition, the nonparametric Mann-Whitney U test was applied to compare data obtained with applied forces lower and higher than 18 nN. The null hypothesis ($H_{0}$) states that, for each viscoelastic parameter ${E_{0}, E_{1},\tau ,\alpha }$, the distributions of values recorded under both regimes share the same central position. The alternative hypothesis ($H_{1}$) posits that these parameters exhibit a significant shift from the central position between the two loading levels. The results of this test show that increasing the indentation force produces a statistically significant change in the four parameters: $E_0$ increases moderately ($p=0.0215$), while $E_1$ and $\tau$ increase strongly and $\alpha$ decreases, all with high significance ($p<10^{-4}$). These values of *p* allow us to reject $H_{0}$ for each case and confirm a systematic dependence of the viscoelastic response on the applied load. To better understand what happens with the parameter $E_{0}$, the only parameter whose significance level was close to the conventional threshold of 0.05, the magnitude of the discrepancy was further quantified using the cosine distance. Specifically, a cosine distance of 0.23 was obtained for this parameter. This value indicates that the two distributions retain considerable overlap and differ only moderately, consistent with the *p*-value of 0.0215; the difference in $E_{0}$ is statistically detectable but of limited magnitude. From a biomedical engineering perspective, the parameter $E_0$ has been associated with the active tension generated by the actin network, which is induced by molecular motors in charge of cell movement and internal organization^79^. Consequently, $E_0$ represents a relevant mechanical indicator for analyzing pathological conditions, such as cancer, where alterations in cell biomechanics play a crucial role in disease progression^52^.

Now, $E_1$ and $\tau$ present statistically significant differences between the two force conditions, as seen in Fig. 13. This indicates a dependence on the conditions used for the measurement, specifically the applied force in this case. The parameter $E_1$, which characterizes the immediate cell elastic response to an applied load, is related to the structural organization of the cytoskeleton on short-time scales. Under low-force conditions ($F < 18$ nN), the mean value of $E_1$ was 3.71 kPa, whereas under high-force conditions ($F > 18$ nN), it increased to 5.33 kPa. This effect is interpreted as an increase in cell rigidity. This increase may be attributed to the involvement of a greater number of cytoskeletal elements in response to increased deformation, as well as strain stiffening. This is a mechanical behavior exhibited by specific cytoskeletal components such as vimentin under compressive or tensile stress^80^, or the simultaneous action of both mechanisms. Similarly, the relaxation time measures the time scale in which the cell reorganizes its internal structure after a mechanical perturbation. A higher value of $\tau$ implies slower reorganization. In our experiments, $\tau$ increased from 1.92 at lower forces to 3.76 at higher forces. In contrast to the other parameters, the fractional order $\alpha$ remains one of the least understood components when obtained from relaxation curves and fitted with a FOZ model. Despite this, our analysis reveals a statistically significant difference between the two force conditions ($p = 1.38 \times 10^{-15}$), suggesting that $\alpha$ may also be related to mechanical stimuli. Specifically, the mean value of $\alpha$ decreases slightly from 0.515 under low force conditions ($F<18$ nN) to 0.504 under high force conditions ($F>18$ nN). This subtle yet significant difference may suggest that $\alpha$ encodes information about the complexity of the relaxation behavior or the organization of the cell’s internal structure. This is because by using different types of force, we obtain different indentations and consequently increase the internal elements involved in the cells.

**Fig. 14:**
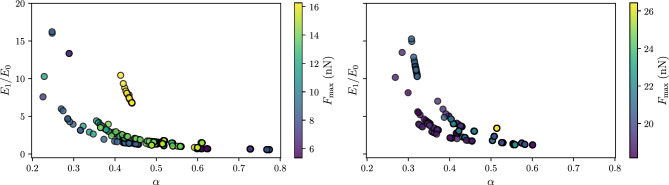
Relationship between the fractional parameter $\alpha$ and the elastic ratio $E_1/E_0$, color-coded by the maximum applied force ($F_{\textrm{max}}$). Measurements for (left) $F < 18$ nN and (right) $F > 18$ nN.

Figure 14 shows the relationship between the fractional exponent $\alpha$ and the elastic ratio $E_{1}/E_{0}$, with individual points colour-coded by the maximum applied force $F_{\textrm{max}}$. The left panel corresponds to low-force experiments ($F<18$ nN), whereas the right panel gathers high-force data ($F>18$ nN). Despite some scatter, both panels display a consistent inverse trend: larger values of $\alpha$ are associated with smaller values of $E_{1}/E_{0}$, indicating a compromise between this ratio and the cell’s fluid-like behavior. The spread in the data also underscores how strongly these viscoelastic parameters depend on experimental details. Sample preparation, temperature, loading protocol, and instrument calibration can all introduce significant variation, so meaningful comparisons between studies require rigorous standardization. Within the AFM community, the SNAP (Standardized Nanomechanical Atomic Force Microscopy Procedure) calibration protocol has demonstrated that systematic adjustment of cantilever sensitivity factors can reduce inter-laboratory scatter in Young’s modulus measurements to about one percent and markedly improve reproducibility for living cells^81^. However, SNAP was designed for the elastic modulus only; parameters that capture time-dependent behavior, such as $\alpha$ and $E_{1}/E_{0}$, remain outside of its scope and therefore demand additional methodological control. Together, these observations suggest the need for comprehensive and standardized measurement procedures. Only with reliable and comparable viscoelastic features will it be possible to advance to the next stage, namely the use of machine-learning models for automatic classification of cellular phenotypes and their degrees of aggressiveness.

## Conclusion

This work presented an integrated approach that combines fractional viscoelastic modeling with automated metaheuristic design to characterize the mechanical response of MG-63 osteosarcoma cells. The proposed framework employed the Fractional-Order Zener (FOZ) model to fit AFM-derived force relaxation curves under different loading conditions, using a tailored metaheuristic ($\text {MH}_*^3$) constructed via a Randomized Constructive Hyper-heuristic with Local Search (RCHH-LS).

The selected configuration, $\text {MH}_*^3$, composed of two swarm-based dynamic operators and one local random walk operator, achieved a performance of $3.00 \times 10^{-3}$, outperforming the worst configuration of the same cardinality by 75.3%. After fine-tuning using Optuna, $\text {MH}_*^3$ reached $2.86 \times 10^{-3} \pm 2.43 \times 10^{-7}$, indicating excellent stability and precision. Moreover, even the worst configuration ($\text {MH}_{\circledast }^3$) improved by 78.8% after tuning, although it remained 42.7% less effective than $\text {MH}_*^3$. These results suggest that while hyperparameter tuning can significantly enhance performance, the original selection of search operators remains the most critical factor.

The generalizability of $\text {MH}_*^3$ was validated on independent AFM datasets, consistently rendering high $R^2$ scores and low error metrics. The viscoelastic parameters extracted ($E_1$, $\tau$, and $\alpha$) exhibited significant sensitivity to the applied force, reflecting the mechanoadaptive behavior of cells, which is likely mediated by cytoskeletal remodeling under increased indentation. In contrast, $E_0$ was the least affected, reinforcing its role as an indicator of intrinsic elastic properties. These results align with those presented in^82^, which explored the effects of different forces using a generalized Maxwell model. In this work, a similar phenomenon occurs; the parameters of $E_1$ and $\tau$ are affected by these variations. Now, in our case, we observe how this effect occurs in the fractional parameter $\alpha$. These findings emphasize the importance of rigorously controlling mechanical loading conditions during experimentation, as variations in force not only influence cell deformation but also directly impact the interpretation of parameters. Neglecting these dependencies may lead to misrepresentations in comparative studies.

Therefore, this work demonstrates that combining fractional viscoelastic modeling with automated algorithm design can yield accurate, robust, and interpretable biomechanical models. The synergy between hyper-heuristics and hyperparameter tuning, known in the literature as Automated Algorithm Design and Configuration, provides a practical path toward generating high-performance solvers without manual intervention, offering valuable tools for computational biomechanics and beyond. Future efforts will focus on refining the AFM acquisition protocol, extending the methodology to additional cell types, and integrating complementary biophysical descriptors. A key priority will be the standardization of these processes, combining computational methodologies and unified measurement protocols to reduce the significant variability currently observed in results. These steps aim to strengthen the robustness of the viscoelastic parameters and broaden the applicability of the proposed framework. Ultimately, we seek to advance toward reliable, data-driven classification of cellular phenotypes.

## Data Availability

The datasets used and/or analysed during the current study are available from the corresponding author on reasonable request. Besides, for repeatability purposes, codes used for this work are freely available at https://github.com/Danielfz14/RCHH-LS_MG63.git.
